# Supplementary material for: Orthotic management of instability of the knee related to neuromuscular and central nervous system disorders: qualitative interview study of patient perspectives
Source: BMJ Open. 2019 Oct 17;9(10):e029313. doi: 10.1136/bmjopen-2019-029313 (PMC6803152; doi:10.1136/bmjopen-2019-029313)
Supplement: Supplementary data [file bmjopen-2019-029313supp001.pdf]

Appendix 1 Interview Topic Guide

Researcher to ask brief details about age, occupation, ethnicity, family members, etc. to frame and contextualise the interview

-Can you tell me a little bit about yourself and how you came to have your orthotic device(s) (such as brace or callipers)? [explore important aspects further, such as diagnosis, referral, care pathway, length of time having an orthosis]

-How has the orthotic device impacted on your daily life?

-[from answer to above question] – What factors have affected you most and why? [explore important aspects further]

-What do you like, or not like, about your orthotic device? Which factors influence your decisions to use your orthotic device and why are they important?

-How effective do you think your device is in the management of knee instability?

-How do you feel about the treatment you have received in connection with your knee instability?

-How could this have been improved?

-What are the goals of treatment that matter most to you?

-How do you feel about the interactions you have had with HCPs? How could these have been improved?

-Any other aspects that have not already been discussed or you would like to expand upon?
