# Supplementary material for: Orthotic management of instability of the knee related to neuromuscular and central nervous system disorders: qualitative interview study of patient perspectives
Source: BMJ Open. 2019 Oct 17;9(10):e029313. doi: 10.1136/bmjopen-2019-029313 (PMC6803152; doi:10.1136/bmjopen-2019-029313)
Supplement: Supplementary data [file bmjopen-2019-029313supp002.pdf]

Appendix 2 Participants' characteristics (study ID, age, gender, condition, duration/severity and reported device use)

Participant	Age (years)	Gender	Condition	Duration/severity/reported device use	Device
P1 (NHS)	36	Female	Poliomyelitis	Since childhood<br>Pain in knee/frequent falls<br>Devices not used as deemed ineffective and ill-fitting	KAFO and AFO
P2 (NHS)	52	Female	Spinal injury	Since childhood<br>Knee pain and instability led to difficulty balancing and falls<br>Awaiting gait assessment and referral to neuro-physiotherapy	Insert in shoe
P3 (NHS)	50	Male	Poliomyelitis	From age 5 years<br>KAFO essential for everyday mobility<br>Uses crutches to walk if not wearing KAFO	KAFO
P4 (NHS)	70	Female	Stroke	Two years previously<br>Hyperextension/pain in knee with limited mobility<br>Brace used to help with mobility but considered ill-fitting	Knee brace
P5 (NHS)	53	Male	Poliomyelitis	Since childhood<br>Severe knee pain, unable to stand for long period due to knee instability, and limited mobility prior to receiving device<br>Device described as 'transformative' in promoting mobility and independence	KAFO
P6 (NHS)	59	Female	Poliomyelitis	From age 16 months<br>Knee instability was primary problem restricting mobility<br>Device essential for mobility	KAFO
P7 (NHS)	55	Female	Multiple sclerosis	Progressive deterioration over ten years<br>Reported trips and falls and foot drop<br>Device mainly used when outdoors to assist with walking	AFO
P8 (NHS)	63	Male	Poliomyelitis	From age 2 years<br>Experienced knee instability/falls during past 5 years<br>Reported KAFO as generally offering good level of support to mobilise	KAFO and AFO

P9 (NHS)	67	Male	Poliomyelitis	From age 5 years<br>Experienced knee instability and falls<br>KAFO viewed as effective and essential for mobilising	KAFO
P10 (NHS)	62	Female	Multiple sclerosis	Diagnosed 30 years previously<br>Instability and weakness in knee and ankle joints, as well as foot drop, resulting in trips and falls<br>Device used mainly to assist with balance and for walking outdoors	AFO
P11 (NHS)	73	Male	Multiple sclerosis	Progressive deterioration in condition over 35 years<br>Weakness and instability in knee, foot drop, and problems with balance<br>AFO deemed effective in promoting mobility	AFO
P12 (NHS)	53	Male	Multiple sclerosis	Diagnosed 32 years previously<br>Marked deterioration in condition over past 8 years<br>Reported having experienced frequent falls, weakness in one knee and foot drop<br>AFO perceived as effective in aiding mobility	AFO
P13 (NHS)	54	Female	Multiple sclerosis	Diagnosed 5 years previously<br>Reported hyperextension of knee and foot drop, resulting in lack of balance and falls<br>Uses AFO and knee brace daily though current knee brace described as ill-fitting	AFO and knee brace
P14 (non-NHS)	64	Female	Poliomyelitis	From age 5 years<br>Reported knee hyperextension, pain and weakness, resulting in frequent trips and falls<br>Recently fitted for KAFO for first time but described it as 'cumbersome' and reported non-use as able to mobilise without it	KAFO
P15 (non-NHS)	72	Female	Poliomyelitis	From age 3 years<br>Experienced weakness in knee joint and problems with balance<br>Alternated use of one NHS-supplied and one privately purchased device	KAFO
P16 (non-NHS)	64	Male	Poliomyelitis	From age 7 years<br>Cannot stand or walk without KAFOs	KAFO (each leg) and spinal brace

P17 (NHS)	72	Male	Spinal injury/drop foot	Four years' duration<br>Difficulty walking on uneven ground<br>AFO perceived to promote mobility and ability to socialise	AFO
P18 (non-NHS)	73	Female	Poliomyelitis	From age 7 years<br>Developed hyperextension of knee 15 years ago and mobility deteriorated<br>KAFO not worn indoors but used for walking outdoors	KAFO
P19 (NHS)	48	Female	Spina bifida; amputation	Spina bifida since birth; date of amputation not reported<br>Severe knee weakness and instability<br>KAFO enables mobility	KAFO
P20 (NHS)	80	Male	Spinal injury	Two years' duration<br>Reported knee pain and instability and loss of balance<br>Brace deemed uncomfortable<br>Intermittent use of brace to assist with walking and employment	Knee brace
P21 (NHS)	58	Female	CMT disease	Progressive condition since birth, with marked deterioration at 30 years of age<br>Foot drop affects both feet<br>AFOs enable mobility outdoors and promote independence<br>Not used in the home	AFO
P22 (non-NHS)	63	Male	CMT disease	Progressive since birth, with marked deterioration at 40 years of age<br>Experienced knee pain and instability and 'weak' ankles, resulting in falls<br>AFOs enable mobility outdoors	AFO
P23 (NHS)	57	Male	Poliomyelitis	From age 3 years<br>Reported loss of balance, knee instability and falls<br>KAFO necessary for mobility	KAFO
P24 (NHS)	63	Male	Poliomyelitis	From age 2 years<br>Knee instability resulting in loss of balance and falls<br>Devices reported as essential for mobility - alternates use of 'new' (privately purchased) and 'old' (NHS supplied) KAFO	KAFO and AFO

Abbreviations: KAFO - knee-ankle-foot orthosis; AFO - ankle-foot-orthosis; CMT-Charcot-Marie-Tooth
