# Supplementary material for: Orthotic management of instability of the knee related to neuromuscular and central nervous system disorders: qualitative interview study of patient perspectives
Source: BMJ Open. 2019 Oct 17;9(10):e029313. doi: 10.1136/bmjopen-2019-029313 (PMC6803152; doi:10.1136/bmjopen-2019-029313)
Supplement: Author's manuscript [file bmjopen-2019-029313.draft_revisions.pdf]

BMJ Open is committed to open peer review. As part of this commitment we make the peer review history of every article we publish publicly available.

When an article is published we post the peer reviewers' comments and the authors' responses online. We also post the versions of the paper that were used during peer review. These are the versions that the peer review comments apply to.

The versions of the paper that follow are the versions that were submitted during the peer review process. They are not the versions of record or the final published versions. They should not be cited or distributed as the published version of this manuscript.

BMJ Open is an open access journal and the full, final, typeset and author-corrected version of record of the manuscript is available on our site with no access controls, subscription charges or pay-per-view fees (http://bmjopen.bmj.com).

If you have any questions on BMJ Open's open peer review process please email info.bmjopen@bmj.com

Orthotic management of instability of the knee related to neuromuscular and central nervous system disorders: a qualitative interview study of patient perspectives

Journal:	*BMJ Open*
Manuscript ID	bmjopen-2019-029313
Article Type:	Research
Date Submitted by the Author:	22-Jan-2019
Complete List of Authors:	Mccaughan, Dorothy; University of York, Health Sciences<br>Booth, Alison; University of York, UK, Department of Health Sciences<br>Jackson, Cath; Valid Research Ltd<br>Lalor, Simon ; Queen Mary's Hospital, St George's University Hospitals NHS Foundation Trust,, Orthotics ; Royal Children's Hospital Melbourne, Orthotics/prosthetics<br>Ramdharry, Gita; Queen Square Centre for Neuromuscular Diseases<br>O'Connor, Rory J.; Univ Leeds<br>Phillips, Margaret Philip; Derby Hospitals/ Nottingham University, Rehabilitation<br>Bowers, RJ; University of Strathclyde, Glasgow, UK, Department of Biomedical Engineering<br>McDaid, Catriona; University of York, York Trials Unit
Keywords:	orthoses, knee instability, patient perspectives, qualitative

SCHOLARONE™
Manuscripts

Manuscript

Orthotic management of instability of the knee related to neuromuscular and central nervous system disorders: a qualitative interview study of patient perspectives

Authors

Dorothy McCaughan[1], Alison Booth[1], Cath Jackson[2], Simon Lalor[3], Gita Ramdharry[4], Rory J O'Connor[5], Margaret Phillips[6], Roy Bowers[7], Catriona McDaid[1]

Institutional addresses

1. Department of Health Sciences, University of York, York, UK
2. Valid Research Ltd., Sandown House, Sandbeck Way, Wetherby, UK
3. Queen Mary's Hospital, St George's University Hospitals NHS Foundation Trust, London, UK
4. Queen Square MRC Centre for Neuromuscular Diseases, National Hospital for Neurology and Neurosurgery & UCL Institute of Neurology, London, UK
5. NIHR Leeds Biomedical Research Centre, Leeds Teaching Hospitals NHS Trust, Chapel Allerton Hospital, Leeds, UK
6. Royal Derby Hospital, Derby, UK
7. Department of Biomedical Engineering, University of Strathclyde, Glasgow, UK

Corresponding author:
Dorothy McCaughan, Department of Health Sciences, University of York, York YO10 5DD, UK
Email: dorothy.mccaughan@york.ac.uk

ORCID iD https://orcid.org/0000-0001-5388-2455

For peer review only

For peer review only - http://bmjopen.bmj.com/site/about/guidelines.xhtml

ABSTRACT

Objectives Adults with knee instability related to neuromuscular disorders or central nervous conditions often experience mobility problems and rely on ortheses to improve function and mobility. Patient views of device effectiveness and acceptability are under-explored. Our study aimed to elicit device users' perspectives regarding acceptability, effectiveness and use of orthoses, and identify important treatment outcomes.

Design Qualitative descriptive study using in-depth semi-structured interviews. Interview transcriptions were coded and thematically analysed, using 'Framework'.

Setting and Participants A purposive sample of 24 adult users of orthotic devices. Nineteen patients were recruited across three geographically dispersed NHS sites, and five people from outside the NHS, through charities/patient support groups across different areas of England. Half of the participants had been diagnosed with poliomyelitis, and the remainder with multiple sclerosis, Charcot–Marie–Tooth (CMT) disease, spinal injury or spina bifida, and stroke. The median age of participants was 64.5 years (range 36–80 years).

Results Participants relied on orthotic devices to enable engagement in daily activities. Patient goals for mobility were closely linked to individual circumstances. Desired treatment outcomes included reduction in pain, trips and falls, with improved balance and stability. Effectiveness, reliability, comfort and durability were the most valued features of orthoses and associated with reported use. Obtaining suitable footwear alongside orthotic devices was a significant concern.

Conclusions Orthotic devices for knee instability play a crucial role in promoting, maintaining and enhancing physical and psychological health and well-being, enabling patients to work, engage in family life and enjoy social activities. Future research should consider how best to measure the impact of orthotic devices on patient quality of life and daily functioning outside the clinic setting, as well as device use and any adverse effects.

This qualitative study is registered as Current Controlled Trials ISRCTN65240228.

Key words: knee instability, neuromuscular conditions, central nervous system conditions, orthoses, patient perspectives, qualitative study

Word count: 3,738 (without quotations); 5,135 (including quotations)

For peer review only

ARTICLE SUMMARY

Strengths and limitations of this study

- This is the first study specifically designed to explore the views and experiences of patients regarding effectiveness and acceptability of orthotic devices, and to identify outcomes that are important for people who have been fitted with an orthotic device for knee instability, across a broad range of conditions.
- Our study captured rich data concerning issues identified as significant by participants, providing important new insights.
- A full range of neuromuscular and central nervous system conditions (for example, myasthenia gravis and muscular dystrophy) were not represented in the sample.
- Nonetheless, the study included people with regular and sustained contact with orthotic services in connection with knee instability.

For peer review only

INTRODUCTION

Adults with knee instability related to neuromuscular disorders (NMD) or central nervous system (CNS) conditions may experience pain, loss of balance, falls and mobility problems. Orthotic devices can offer support, align or correct deformities, or improve function.[1,2] A knee–ankle–foot orthosis (KAFO) can be prescribed when other forms of bracing, such as an ankle–foot orthosis (AFO) or knee orthosis (KO), are insufficient to adequately control knee instability, due to weakness or muscle imbalance. However, usage can be low.[3-5] Two recent surveys of orthoses users in Belgium[2] and Malawi and Sierra Leone[6] report varying levels of satisfaction with devices, in part related to differences in service delivery in each country. While inclusion of ‘open’ questions in both surveys elicited ‘free text’ responses from participants, the underlying reasons for device satisfaction and use were not explored in depth, as this requires the use of appropriate qualitative methods. A focus group with eight AFO users living in the Netherlands to gain insight into importance of device related activities to AFO-users found that participants ranked walking as the most important of 11 different activities.[7] However, this small-scale study did not include any KAFO users, whose priorities for treatment outcomes may differ.

Understanding of the treatment outcome priorities of people with knee instability related to neuromuscular and CNS conditions is limited, and the range of different outcome measures used to evaluate patient outcomes following device fittings[8,9] makes it difficult to draw conclusions about effectiveness. Our study aimed to: (i) explore patient perceptions regarding acceptability, effectiveness and usage of orthoses, and (ii) identify the outcomes that are important for people who have been fitted with an orthotic device for knee instability, across a broad range of conditions. The qualitative study reported here comprises one strand of a larger study.[10]

METHODS

Study design

For peer review only - http://bmjopen.bmj.com/site/about/guidelines.xhtml

Semi-structured individual interviews were undertaken (during 2014-2015) with adult users of orthotic devices. Qualitative approaches are well-suited to investigation of phenomena about which little is known, while semi-structured interviews offer flexibility in data collection and generate rich narratives, allowing the researcher to analyse how participants make sense of the topic under investigation.[11] We followed the Consolidated Criteria for Reporting Qualitative Studies guidelines for reporting qualitative research.[12]

Patient and public involvement
The study was designed in collaboration with a patient advisor, herself a user of an orthotic device, who contributed to the design and piloting of the interview topic guide (Appendix 1) and with interpretation of the study results.

Sampling
Participants were purposively sampled[13] to reflect a range of conditions, ages, gender, length of time fitted with an orthosis, and regions across England. Twenty-four people participated, nineteen recruited through NHS clinics and five through charities/patient support groups. Participants were adults diagnosed with a neuromuscular or CNS condition who had been offered an orthotic device for knee instability. Twelve had been diagnosed with poliomyelitis, and the remainder with multiple sclerosis, Charcot–Marie–Tooth (CMT) disease, spinal injury, stroke and spina bifida. Participants' ages ranged from 36 to 80 years (median 64.5 years). Half worked part or full-time while the remainder were retired; most (18) lived with their spouse and/or other family members.

Data collection
An interview topic guide was developed from existing literature, experiences of the research team and input from the patient advisor. Two experienced qualitative researchers (DM and CJ) undertook the interviews. Twenty-one interviews were conducted in participants' homes; three people requested a telephone interview as this was more convenient. In two cases, family members were present during the interview and their comments were included in data analysis. Interviews lasted approximately one hour and all were audio-recorded. Data collection continued until no further pertinent information was forthcoming (after 24 interviews). Audio-recordings were fully transcribed and a random sample of six transcripts checked for accuracy.

Data analysis
Data were analysed for thematic content using the 'Framework' method[14] (see figure 1). Framework is a flexible tool, not aligned with a particular epistemological, philosophical, or theoretical stance, but adaptable to various qualitative approaches aiming to generate themes. NVivo software package[15] facilitated data handling and retrieval and enabled comparison within and between cases. Analysis was systematic and iterative[16] and involved close reading and detailed coding of transcripts. To promote analytical rigour, coding was cross-checked in four early transcripts by the two experienced qualitative researchers (DM, CJ) involved in the study. We sought to identify commonalities and differences in the data, with the aim of drawing descriptive and/or explanatory conclusions clustered around themes.[17] Differences in identification and development of themes were discussed until consensus was reached, and the thematic framework accordingly modified and

For peer review only - http://bmjopen.bmj.com/site/about/guidelines.xhtml

expanded to accommodate all participant responses across the data set. Examination of negative or ‘deviant’ cases[18] helped to develop and refine the analysis. Contributions from the patient advisor promoted reflection and interpretation of the study findings.

RESULTS

Five principal themes were identified: (1) impact of NMD and CNS conditions on walking and mobility; (2) fitting/acquisition and use/wearing of orthotic devices; (3) perceived positive and negative aspects of orthotic devices; (4) footwear; (5) desired treatment goals and outcomes. These themes are described below. Details relating to further themes/subthemes can be found in the main report.[10]

1. Impact of NMD and CNS conditions

Participants’ mobility was impaired by a range of symptoms and sequelae (see Box 1) associated with their condition, including pain, muscle weakness, fatigue, loss of balance, unsteady gait, and frequent trips and falls.

Box 1 Symptoms and sequelae associated with NMD and CNS conditions

Reported symptoms
-Pain (sometimes severe) in knee or ankle joints
-Hyperextension of knee joint
-Muscle weakness in the lower limb and/or of the muscles supporting joints
-Limbs of unequal length
-Feet ‘frozen’ in abnormal position
-Varying degrees of paralysis in lower limbs and feet
-Toes that ‘curl inwards’
-Drop foot
-Fatigue

Reported sequelae
-Frequent falls, often preceded by feeling that leg is about to ‘give way’ under them
-Loss of sense of balance and stability
-Lop-sided gait
-‘Dragging’ of feet
-Frequent trips, especially on uneven surfaces
-Difficulty standing for long or walking any but short distances
-‘Wear and tear’ in unaffected or ‘good’ limb due to transfer of weight and effort in walking

Limited mobility was associated with feelings of fearfulness and anxiety, resulting in diminished self-confidence and independence. People in employment were particularly anxious to gain or retain mobility as far as their condition allowed. Many participants described being proactive in trying to postpone or abate the impact of future deterioration on independent mobility, by keeping their weight under control, exercising regularly, and undertaking exercise programmes recommended by physiotherapists. Reduced mobility was said to limit the pursuit of enjoyable activities such as gardening and walking, and could lead to social isolation. Retaining the ability to drive was viewed as important for both work and leisure purposes.

For peer review only - http://bmjopen.bmj.com/site/about/guidelines.xhtml

'The last time I was on the tube I got pushed…and I lack confidence and get scared, so if I haven't got somebody with me, I won't use the tube.' (Participant (P) 23)

'I was in a very well paid job… and I saw how much I deteriorated in the last 5 years. . . I try to help myself…like with my weight… I have to work on that and my exercise . . . I am active.' (P22)

'Obviously I walk slower, so I am on my own quite a bit because I have got good friends who will walk with me, but a lot of people [won't] . . . so it does actually make you an alone sort of person' (P2)

'I've got an automatic car . . . I've got a mobility car with a hoist in the back which can pick up my scooter.' (P18)

Unsurprisingly, obtaining the 'right' orthotic device(s) to help with current and future mobility emerged as a central concern of all study participants.

2. Fitting/acquisition and use/wearing of orthotic devices

Fitting/acquisition of knee–ankle–foot orthoses (KAFOs)

Participants who used a KAFO (n=12) all had a diagnosis of poliomyelitis. These participants emphasised the importance of having a 'spare' KAFO for use during the period of being fitted for, and 'breaking in', a new device. Fitting was frequently described as a slow process, involving numerous appointments, often spaced at long intervals, and sometimes cancelled at short notice by the service provider. Attending appointments could mean time away from work, due to lack of 'out of hours' appointments, and some people mentioned difficulties with transportation. The fitting of a new device could be particularly challenging for those with complex condition-related problems; some individuals recalled attending multiple appointments stretching across a 2-3 year period before finally obtaining a device that fully met their needs and was comfortable.

'If you're not getting the appointments, you're not getting the work done' (P19)

'It was not a standard case, it made it much harder and I think for those first 2 or 3 years where it seemed I was going backwards and forwards to [clinic] and I felt that no real progress was being made' (P5)

Many people mentioned the importance of having sufficient time during fitting appointments. 'Double' appointments were said to allow more time for assessment and minor adjustments to devices that promoted optimal fit. Time pressures were perceived as constraining orthotist-patient communication regarding the new device and any adaptations to gait that might be needed. Problems might only come to light after the patient returned home and they could wait some considerable time for another appointment, unable to use the issued orthosis in the interim. Adequate time for full discussion during appointments was highlighted as particularly important to people receiving a KAFO for the first time, who might be struggling to accept and adapt to their orthosis.

'You don't really sort of pick up that sort of a problem when you're having a short appointment…you're only there for 20 minutes, half an hour…you only think about the questions afterwards' (P14)

For peer review only - http://bmjopen.bmj.com/site/about/guidelines.xhtml

A minority of participants reported receiving a device so ill-fitting that it was unwearable; participant 15, for example, reported seeking a new KAFO through private (non-NHS) suppliers due to the poor fit of their NHS device.

'It wasn't a question of being uncomfortable, it didn't fit. It just did not fit and they took it away – I didn't even leave with it.' (P8)

'It was too big, too high, so it was going right into my buttocks, it was too wide, so I wasn't getting support at the knee and it was like it wasn't made for me.' (P15)

Participants underscored the importance of being fitted for new footwear (discussed below) at the same time as being fitted for a new KAFO.

'every calliper kind of needs a new boot to kind of fit it, so I was wearing the old calliper and had to wear it with these boots which didn't kind of work with the calliper.' (P23)

Fitting/ acquisition of ankle-foot orthoses (AFO) and knee braces

Four of the five AFO users were satisfied with the fit of their device. The fifth described her new AFOs as *'too painful to wear'* and causing damage to her skin, and she reverted to using her 'old' devices while waiting for the new ones to be adjusted.

'I've just had a new pair . . . at the moment I've been bedding them in but something is not quite right with them . . . I've worn them for about a month but my foot has started to hurt again and I think the padding is not quite in the right place…' (P21)

Two people (one diagnosed with a spinal injury, one with stroke) had been supplied with 'off the shelf' knee braces through physiotherapy services, but found them ill-fitting and uncomfortable, so that they remained unused.

'I just couldn't bear it. When I walked it just dug in, you can see the bruising….I would have preferred a bespoke one right from the start' (P4)

Use/wearing of orthotic devices

Device usage ranged along a continuum, from full-time use during the day, to little, or no use. Usage was closely linked by participants to capacity for independent mobility and therefore crucial to daily life. Many described their orthosis as integral to their social identity, enabling them to fulfil their desired roles as employee, spouse/partner, parent, and friend.

'I need my surgical appliance to get on with my life . . . it needs to be effective and reliable . . . I want to be in the real world. I love it . . . I'm in a working world and I just want to get on with it' (P2)

Consequently, device malfunction was highly disruptive to 'normal' life.

'if it breaks, you're kind of thrown really . . . I came out of work, it was about half past six, and I got across the road and the calliper just went, it just snapped' (P6)

For peer review only - http://bmjopen.bmj.com/site/about/guidelines.xhtml

'As soon as this thing (KAFO) breaks down, I'm in silly street . . . that's not a place I want to go' (P19)

Other people reported using their device depending on the circumstances; for example, using their device outdoors, but not when at home. Intermittent use was also reported during *'breaking in'* of a new device, including alternating between wearing the new and 'old' devices. Reluctance to make the transition from an 'old' to a new device was widespread, due to '*teething troubles'* with the new device, and/or because of the need for gait adjustment, described by participant 18 as *'like having to learn to walk again'.*

Three of the 24 participants reported almost no use of their device as it did not offer adequate joint support. Additionally, a poor fitting device could result in skin damage and/or pain and discomfort, resulting in non-use. The most extreme (and therefore atypical) report of receipt of an ill-fitting orthosis, came from Participant 1 (diagnosed with poliomyelitis in childhood), who commented that she had never worn any of numerous orthoses provided for her, as they had been ineffective, ill-fitting and uncomfortable to wear. During interview, participant 1 retrieved from elsewhere in the house the KAFO that she had most recently received through orthotic services, still in its plastic wrapper, and described by her as *'a complete waste of NHS money'.* Instead, she preferred to rely on a stick to help her mobilise, while recognising that this was not a good long-term solution, and she said she was finding it increasingly difficult to mobilise.

3. Perceived positive and negative aspects of orthotic devices

Unsurprisingly, participants' perspectives of positive and negative aspects of their orthosis (see figure 2) synchronised with their reports of device use or non-use. Functionality and reliability were overwhelmingly considered more important than cosmesis, though appearance of the device was a significant feature for some of those interviewed (both male and female), who described the *'ideal'* device as light, sleek and discreet.

Aspects of devices viewed positively

Effectiveness of the device to control pain and offer support for the knee or ankle, and/or to assist with lifting the foot, alongside reliability and durability, were the features viewed most positively. Reliability of the device to withstand wear and tear, was highly valued. The people most satisfied with their orthosis referred to the *'transformative'* effects on their lives of a well-fitting and well-functioning device.

'it's [KAFO] *really transformed my life….from here to [place name] may not appear sort of a long distance but for me I don't think I could have done it beforehand, now I can which is a major sort of step forward . . . it's made me much more mobile than I ever was before really'* (P5)

'I was frightened to go out . . . It's just like, they [AFOs] just give me a new lease, you know, they give me my independence to go out on my own…with these splints I've got my independence. I can go out without my husband, I can go out with my friends.' (P21)

Appearance, light-weight, and 'user-friendliness' (the ease with which the device could be donned and doffed, fastenings that are easy to manage and reliable, ease

For peer review only - http://bmjopen.bmj.com/site/about/guidelines.xhtml

of use with preferred clothing, and 'breathability of the materials used) were secondary concerns, though also appreciated.

'he showed me one at the time, which looked pretty cool . . . it just looked really, really good.' (P8)

'It has carbon fibre, so it's lighter…it's not as wide, I can use most trousers' (P3)

'modern day materials are absolutely fabulous . . . there is no weight in them and they are very strong' (P22)

'It's speedy . . . you can put it on quickly' (P6)

Aspects of devices viewed negatively

Devices providing inadequate knee support, that were poor fitting (resulting in pain, discomfort or skin damage), and/or prone to breakage or malfunction, were described in strongly negative terms.

'this doesn't stay on my knee, as I am walking it falls right to the bottom. . as I am walking it's slipping down…If it worked, it would be OK . . . there's no benefit in it…it just doesn't do nothing . . . I swear, I feel completely hopeless' (P1)

'we went to [city] and literally we'd just landed at [name] airport and I'm walking to get my suitcase and I fell flat on my face . . . the thingy here, see there, it just went. Really is this screw holding me, I mean can't you think of something stronger than that! . . the whole thing is very basic… the holiday was ruined…I couldn't relax… it was a short trip.' (P23)

Breakage or device malfunction represented a crisis, temporarily depriving patients of their independence and interfering with plans and activities. Having a spare device in this situation was important to the majority of patients. They pointed out that even a seemingly simple repair, such as having leather straps replaced when they wear out, could result in difficulties and inconvenience. Other concerns related to discomfort, due to the device being heavy, bulky or cumbersome, devices considered aesthetically displeasing, difficult to put on or take off, and those that caused damage to clothing and/or footwear. Dislike of hook-and-loop fastenings was common; they did not always hold the device securely, could 'snag' on tights, and collect fabric fibres and fluff making them ineffective. Restrictions to choice of clothing and/or footwear to accommodate devices could be irritating; three male participants reported wishing to appear smartly dressed on formal business or social occasions, but having to wear wide-legged trousers to accommodate their device.

'I have buckles, I hate Velcro' (P16)

'it's difficult to get trousers that are really wide . . . so I buy women's jeans . . . from second-hand shops, the charity shops . . . callipers damage them, I can't pay £50–£70' (P16)

Serious skin damage in relation to wearing a KAFO or AFO was rarely reported, though marking or bruising of the skin in connection with newly acquired and/or ill-fitting devices was commonly mentioned, particularly where the knee is in close proximity to the joint on the KAFO. Skin rashes were mainly associated with plastic devices. Examples of 'home grown' measures taken to prevent discomfort and minor skin damage included wearing tights, leggings or pyjamas under the device, adding

For peer review only - http://bmjopen.bmj.com/site/about/guidelines.xhtml

extra padding at pressure points or, in one case, buying ankle straps that were made of softer leather than those supplied. Warm weather could give rise to skin irritation and several people mentioned looking forward to taking their device off at the end of the warm day to allow their skin to *'breathe'.*

'During the summer I quite often wear pyjama bottoms underneath the straps and leather of the calliper' (P9)

'One year…it was very hot… and I didn't have any tights on… it just rubs your skin, you can't wear it against the skin… I had blisters' (P18)

'It's a bit like taking your shoes off . . . when I take the calliper off the skin can breathe a bit more' (P3)

4. Footwear

Being fitted for new shoes at the same time as for a new orthosis was considered important and positive and negative aspects of footwear were mentioned by participants (see figure 3). KAFO users diagnosed with poliomyelitis explained they might require each shoe of a pair to differ in width or height as their specific requirements can change over time, and require frequent re-assessment.

'I had to insist and say, look none of your shoes is fitting me properly . . . I haven't had them reviewed since the 90s and I'm a lot older . . . every time I come, something is wrong.' (P15)

Delays to the manufacture and delivery of new shoes were experienced as frustrating and disappointing. Shoes were said to require frequent repair as heels and soles can wear out quickly. Participants disliked having to rely on one pair of shoes for everyday use while repairs were being carried out on their other pair; many people said they usually owned only two pairs of orthotic shoes at any one time, regarding two pairs as their maximum entitlement. A good fitting shoe that avoided damage to skin or deeper tissues were priorities. Comfort was generally valued over appearance, although many people expressed a preference for footwear described as *'discreet'*. Heavy or *'clumpy'* orthotic shoes were said to interfere with ease of walking, and to draw attention to their condition. Several participants commented that the grip on the soles of shoes supplied to them was often inadequate for walking on uneven, muddy or icy surfaces, and suggested that a walking-shoe type of sole could improve confidence with walking outdoors.

'there is no point in weeping over kitten heels because I am never going to wear those. But something reasonably presentable, that is not too intrusive, because you've got enough to be getting on in life with without having some people staring at things you might be wearing because obviously I am a bit conscious of the way I do walk . . . and people do stare a bit, so something that can be quite discreet is important.' (P2)

Having a wide choice of colour, fastening (some people found hook-and-loop easier to use than laces), material (leather or suede), and style of shoes, was appreciated, and most people thought that choice and quality of orthotic footwear have improved in recent years. Several people mentioned usually choosing black or brown shoes to '*go with anything'* and as suitable for work, while a few thought these were the only

For peer review only - http://bmjopen.bmj.com/site/about/guidelines.xhtml

colours available to them. One person insisted on brightly coloured shoes to suit his personality.

'Because of my personality, I like bright shoes, for example, I always wore bright green shoes . . . these are quite bright red really…it is part of your identity.' (P5)

Participants using other types of orthoses (such as a knee brace) reported that they usually purchased shoes from the high street, preferring particular brands of 'sturdy' shoes. Other people reported using orthotic devices such as insoles and inserts inside 'normal' shoes, either supplied through orthotic services or bought from commercial suppliers.

5. Desired treatment goals and outcomes

Effective support for the affected (knee or ankle) joint with concomitant reduction of joint pain was the prime goal for most participants. Individuals wished to be as confidently mobile as their condition would allow, achieved through reduced anxiety that their joint might suddenly *'give way',* and avoiding trips and falls. Preventing future deterioration of mobility (as far as feasible in relation to their specific condition) was a significant long-term goal. It was clear from participants' accounts that their desired treatment goals related to both physical and psychosocial aspects of their everyday lives. Cited benefits of independent and safe mobility included increased employment opportunities, ability to participate in day-to-day family activities, and social events with friends and colleagues, as well as independent travel, and regular exercise, all regarded as vital to current and future physical and mental well-being.

'I just do not want to become housebound, so that primarily is the objective of my orthotics.' (P22)

'I need my surgical appliance to get on with my life . . . it needs to be effective and reliable' (P2)

'It's just enabling me to keep going as long as possible which is really important to me.' (P10)

'Walking is essential to him . . . to keep active as much as possible.' (Wife of P9)

Many people emphasised being able to continue to drive their car as vital for work and leisure.

'It's very important having a car, I couldn't do my job without a car' (P3)

'I want to drive down and pick my mum up and bring her here' (P10)

Most study participants seemed to have accepted that they were unlikely to ever be able to walk very fast, or for very far, without some degree of fatigue, and they showed little interest in increasing the speed of their walking, or distance covered, as treatment goals per se. Instead, goals were expressed in terms of enablement of mobility, within the context and confines of individual circumstances, such as being able to work full-time and undertake world-wide travel, walk a few hundred metres to nearby shops, or being able to take just a few steps.

'My walking is so limited. If I can stand and get something out of a cupboard and walk a few steps and get back to the wheelchair or whatever, that's what I can do, and that's what I need to do. For

For peer review only - http://bmjopen.bmj.com/site/about/guidelines.xhtml

example in [name] mother's house I can't get the wheelchair in . . . because of the threshold, so I have to get out, walk a couple of steps over the threshold and then get back in the wheelchair and to be able to just walk those few steps to stand is an enormous advantage.' (P16)

DISCUSSION

Principal findings

Effectiveness, reliability, comfort and durability were the most valued features of orthoses, and seemingly closely linked to their use, while cosmesis was perceived as of secondary importance. Reduction in pain, trips and falls, and improved balance and stability, promoting independent mobility were the most important outcomes to participants. The standard used by many participants to assess the effectiveness of their device was the extent to which it enabled them to engage in a broad range of activities viewed as important for physical and mental well-being. For some, even limited independent movement was regarded as a valued outcome. Comfort (fit and weight) of shoes, appearance, and availability of choice regarding colour, materials, types of fastenings and style were all considered important by participants in our study, factors that seemed to be associated with likely wearing of prescribed shoes.

Results in the context of other studies

As part of the same project we also undertook a systematic review of the evidence on effectiveness of orthotic devices for the management of knee instability in adults with a neuromuscular or CNS disorder.[19] The review found that effectiveness studies focused on outcomes such as gait quality and energy consumption, assessed in the clinic setting. While important outcomes, this focus neglects many outcomes identified as important by participants in this qualitative study.

Our findings align with Schaffalitzky et al.'s[20] study with 24 lower limb prosthetic users that revealed that even small gains in mobility provided by a device may constitute a successful outcome from the patient's perspective. Evidence relating to acceptability and use of KAFOs is limited. Swinnen et al.[2] have reported device functionality and comfort as more highly valued than appearance amongst 33 patients with neurological conditions (28 of whom used an AFO), views also widely held by participants in our study. Our study findings also echo results from previous studies of patients' views of AFOs[21-23] indicating that orthoses that were ill-fitting, considered uncomfortable, heavy, cumbersome and unsightly, and which drew attention to disability, were less acceptable to patients.

The important contribution of footwear to people's self-image, identity and self-expression is well recognised [24,25] and was a significant concern for both male and female participants in our study, although good fit and comfort were prioritised.

Participants in our study repeatedly emphasised the need for individually tailored care, indicating the need for a service model such as the one advocated by Hutton and Hurry,[26] where orthotic products are not viewed as commodities, but as individually prescribed solutions suited to each patient's personal needs. Lengthy waiting times to access orthotic services and frequent delays in the provision and repair of custom-made KAFOs and footwear were commonly reported in our study, as elsewhere[26-29] as impeding device use. Our study participants also emphasised the need for intensive support when receiving a device for the first time, as they

For peer review only - http://bmjopen.bmj.com/site/about/guidelines.xhtml

adjust to altered self-image. They suggested that in addition to technical information, patients may require psychological support, which has been highlighted previously.[30,3]

Strengths and weaknesses of the study
Our study used robust methods, including development of the topic guide with a patient and clinicians, and quality assurance of data collection, coding and thematic analysis. A full range of neuromuscular and CNS conditions (for example, myasthenia gravis and muscular dystrophy) are not represented in the sample. Nonetheless, the study included people with regular and sustained contact with orthotic services in connection with knee instability. In addition, we recruited sufficient patients to achieve data saturation,[31] within the limits recommended by Hennink et al.[32] to achieve *'meaning saturation'*, which enables a richly textured understanding of issues. Inclusion of different age groups revealed some differences in perspectives. Whereas younger participants in our study emphasised the importance of their orthosis in enabling them to undertake full-time employment and function in the role of family breadwinner, older (often retired) people often talked about device use in facilitating their involvement in family and leisure activities that helped them feel connected to the physical and social world beyond their home. Owing to the comparatively under-investigated nature of our research topic, generalisability of our study findings may be less salient than 'sensitising' readers to new information,[16] captured through in-depth interviews, promoting new ways of thinking about patients' perspectives of using orthotic devices.

Applicability to clinical practice and suggestions for further research
The findings have implications for the training of healthcare professionals involved in the prescribing, fitting and follow-up of patients using orthotic devices who have complex conditions. To maximise patient acceptance and use of prescribed devices, it is important that the relevant healthcare professionals have the appropriate skills, and the necessary time during consultations, to establish a relationship where patients feel listened to, and that supports patients to identify acceptable management strategies and achieve desired treatment outcomes.

Development and use of a core set of patient-reported outcome measures in the clinical and research setting would facilitate assessment of the effectiveness of different devices and management strategies. Brehm et al.[33] have suggested development of a core set of outcomes for studies of lower limb orthoses, based on the World Health Organization International Classification of Functioning Disability and Health.[34] Our research highlights important aspects that should be included from the patient experience, such as quality of life and psychological well-being, as reflected through ability to engage in a broad range of activities viewed as important for physical and mental well-being, and reduction in pain, trips and falls. It is important that patients are involved in any future work to develop a core outcome set.

CONCLUSION

Orthotic devices for knee instability play a crucial role in promoting, maintaining and enhancing physical and psychological health and well-being, enabling patients to work, engage in family life and enjoy social activities. Reduction in pain, trips and falls, and improved balance and stability, linked to potential for independent mobility,

For peer review only - http://bmjopen.bmj.com/site/about/guidelines.xhtml

were regarded as important treatment outcomes. Future research should consider how best to measure the impact of orthotic devices on patient quality of life and daily functioning outside the clinic setting, as well as device use and any adverse effects.

Author affiliations

1. Department of Health Sciences, University of York, York, UK
2. Valid Research Ltd., Sandown House, Sandbeck Way, Wetherby, UK
3. Queen Mary's Hospital, St George's University Hospitals NHS Foundation Trust, London, UK
4. Queen Square MRC Centre for Neuromuscular Diseases, National Hospital for Neurology and Neurosurgery & UCL Institute of Neurology, London, UK
5. NIHR Leeds Biomedical Research Centre, Leeds Teaching Hospitals NHS Trust, Chapel Allerton Hospital, Leeds, UK
6. Royal Derby Hospital, Derby, UK
7. Department of Biomedical Engineering, University of Strathclyde, Glasgow, UK

Acknowledgements We gratefully acknowledge all the participants who took part in interviews. We would especially like to thank our patient advisor, Ms Sam Bunting, in her role as patient representative. We also wish to acknowledge the contribution of Lisa Dyson to conception of the study.

Author Contributions CMcD, AB, RB, GR, SL and MP designed the study. RJOC provided clinical advice. DM and CJ recruited participants and conducted interviews. Transcripts were coded and analysed by DM with input from CJ. DM wrote the first draft of the manuscript, which was revised with CMcD and AB. All authors commented on and approved the final manuscript.

Funding This project was funded by the National Institute for Health Research (NIHR) HTA Programme (project number 13/30/02) and has been published in full in Health Technol Assess 2016;20(55). Further information available at https://www. journalslibrary. nihr. ac. uk/ programmes/ hta/ 133002/#/ This report presents independent research commissioned by the NIHR. The views and opinions expressed by authors in this publication are those of the authors and do not necessarily reflect those of the NHS, the NIHR, MRC, CCF, NETSCC, the HTA programme or the Department of Health.

Competing interests During this study, SL was an employee of Opcare, a company that provides orthotic and prosthetic services to the UK NHS. This company does not manufacture orthotic devices, although a sister company ORTHO C FAB does.

Patient consent for publication Not required.

Ethics approval The study received research ethics (REC reference 14/LO/1132) and governance approvals to recruit patients through NHS sites. The University of York's Department of Health Sciences' Research Governance Committee gave approval to recruit study participants via patient support groups/charities. Written consent was obtained prior to interviews. Participants were given assurances

For peer review only - http://bmjopen.bmj.com/site/about/guidelines.xhtml

concerning confidentiality and anonymity and informed that they could withdraw from the study at any time.

Provenance and peer review Not commissioned; externally peer reviewed.

Data sharing All data and material related to this research are archived and maintained according to organisational and ethical regulations. Data are not publicly available due to the risk of participant identification from specific contexts revealed when reading entire transcripts and due to the terms and conditions regarding the release of data to third parties upon which ethical approvals for this study were contingent. Reasonable requests for further information relating to data can be made to the corresponding author.

REFERENCES

1. Ramdharry G, Pollard A, Marsden J, et al. Comparing gait performance of people with Charcot-Marie-Tooth disease who do and do not wear ankle foot orthoses. *Physiother Res Int* 2012; 17:191–9.

2. Swinnen E, Lafosse C, van Nieuwenhoven J, et al. Neurological patients and their lower limb orthotics: An observational study about acceptance and satisfaction. *Prosthet Orthot Int* 2017; 4(1):41-50.

3. Vinci P and Gargiulo P. Poor compliance with ankle-foot-orthoses in Charcot-Marie-Tooth disease. *Eur J Phys Rehabil Med* 2008; 44:27–31.

4. Chisholm AE and Perry SD. Ankle-foot orthotic management in neuromuscular disorders: recommendations for future research. *Disability* 2012; 7:437–49.

5. Michael JW. KAFOs for ambulation: an orthotist's perspective. *J Prosthet Orthot* 2006; 18:187–91.

6. Magnusson L and Ahlstrom G. Patients' Satisfaction with Lower-limb Prosthetic and Orthotic Devices and Service delivery in Sierra Leone and Malawi. *BMC Health Serv Res* 2017; 17:102.

7. Van der Wilk D, Hijmans JM, Postema K, et al. A user-centred qualitative study on experiences of ankle-foot orthoses and suggestions for improved design. *Prosthet Orthot Int* 2018; 42:121-128.

8. Hebert JS. Ambulatory KAFOs: a physiatry perspective. *J Prosthet Orthot* 2006; 18:169–74.

9. Peaco A, Halsne E and Hafner BJ. Assessing satisfaction with orthotic devices and services: a systematic literature review. *Am Acad Orthot Prosthet* 2011; 23(2): 95-105.

10. O'Connor J, McCaughan D, McDaid C, et al. Orthotic management of instability of the knee related to neuromuscular and central nervous system disorders:

For peer review only - http://bmjopen.bmj.com/site/about/guidelines.xhtml

systematic review, qualitative study, survey and costing analysis. *Health Technol Assess* 2016; 20 (55).

11. Pope C and Mays N. (eds.). *Qualitative research in health care*. 3rd ed. London: BMJ Books, 2006.

12. Tong A, Sainsbury P, Craig J. Consolidated criteria for reporting qualitative research (COREQ): a 32-item checklist for interviews and focus groups. *Int J Qual Health Care* 2007;19:349-57.

13. Patton MQ. *Qualitative Research and Evaluation Methods: Integrating Theory and Practice.* 4th ed. Thousand Oaks, California: Sage Publications, 2015.

14. Gale NK, Heath G, Cameron E, et al. Using the framework method for the analysis of qualitative data in multidisciplinary health research. *BMC Med Res Methodol* 2013; **13**:117

15. Richards L. *Handling Qualitative Data: A Practical Guide.* London: Sage, 2005.

16. Green J and Thorogood N. *Qualitative Methods for Health Research*. 4th ed. London: Sage, 2018.

17. Harding J. *Qualitative data analysis.* 2nd ed. London: Sage, 2019.

18. Pope C, Ziebland S and Mays N. Analysing qualitative data. *BMJ* 2000;320 (7227):114-116.

19. McDaid C, Fayter D, Booth A, et al. Systematic review of the evidence on orthotic devices for the management of knee instability related to neuromuscular and central nervous system disorders. *BMJ Open* 2017;7:e015927. doi:10.1136/ bmjopen-2017-015927

20. Schaffalitzky E, Gallagher P, Maclachlan M, et al. Understanding the benefits of prosthetic prescription: exploring the experiences of practitioners and lower limb prosthetic users. *Disabil Rehabil* 2011; 33:1314–23.

21. Phillips MF, Radford K, Willis A. Ankle foot orthoses for people with Charcot Marie Tooth disease: views of users and orthotists on important aspects of use. *Disabil Rehabil Assist Technol* 2011; 6:491–9.

22. Fisher LR and McLellan DL. Questionnaire assessment of patient satisfaction with lower limb orthoses from a district hospital. *J Prosthet Orthot* 1989;13:29–35.

23. Bulley C, Shiels J, Wilkie K, et al. User experiences, preferences and choices relating to functional electric stimulation and ankle foot orthoses for foot drop after stroke. *Physiotherapy* 2011; 97(3): 226–33.

24. Williams A, Nester C, Ravey M. Rheumatoid arthritis patients' experiences of wearing therapeutic footwear: a qualitative investigation. *BMC Musculoskelet Disord* 2007; 8:104.

For peer review only - http://bmjopen.bmj.com/site/about/guidelines.xhtml

25. Van Netten JJ, Francis A, Morphet A, et al. Communication techniques for improved acceptance and adherence with therapeutic footwear. *Prosthet Orthot Int* 2017; 41(2):201-204.

26. Hutton J, Hurry M. Orthotic Service in the NHS: Improving Service Provision. Report, University of York, York Health Economics Consortium, 2009.

27. Audit Commission. Fully Equipped: The Provision of Equipment to Older or Disabled People by the NHS and Social Services in England and Wales. London: Audit Commission, 2000.

28. Hill ME and Phillips MF. Service provision for adults with long-term disability: a review of services for adults with chronic neuromuscular conditions in the United Kingdom. *Neuromuscul Disord* 2006;16:107–12.

29. NHS England. Improving the Quality of Orthotic Services in England. 2015. https://www.england.nhs.uk/commissioning/wp-content/uploads/sites/12/2015/11/orthcs-final-rep.pdf (2015, accessed 16 November 2018)

30. Garralda ME, Muntoni F, Cunniff A, et al. Knee-ankle-foot orthosis in children with Duchenne muscular dystrophy: user views and adjustment. *Eur J Paediatr Neurol* 2006;10:186–91.

31. Saunders B, Sim J, Kingstone T, et al. Saturation in qualitative research: exploring its conceptualization and operationalization. *Qual Quant* 2018; 52:1893–1907

32. Hennink MM, Kaiser BN and Marconi VC. Code saturation versus meaning saturation: how many interviews are enough? *Qual Health Res* 2017; 27(4): 591-608.

33. Brehm M, Bus SA, Harlaar J, et al. A candidate core set of outcome measures based on the international classification of functioning, disability and health for clinical studies on lower limb orthoses. *Prosthet Orthot Int* 2011; 35(3) 269–277.

34. World Health Organization (WHO) International Classification of Functioning Disability and Health: ICF 2001; Geneva: World Health Organization.

Figure Legends/captions

Appendix 1 Topic Guide
Figure 1 Data analysis using Framework approach
Figure 2 Study participants' perceptions of positive and negative aspects of their orthoses
Figure 3 Study participants' perceptions of positive and negative aspects of footwear

For peer review only - http://bmjopen.bmj.com/site/about/guidelines.xhtml

Figure 1 Data analysis using ‘Framework’ approach

Familiarisation with data
Reading and re-reading transcripts

Identification of codes
Identification of codes (units of meaning)
Coding scheme developed and applied to all transcripts
Data attributed to individual codes

Identification of themes
Codes combined/expanded to develop themes (broader categories of meaning)

Reviewing of themes
Iterative, cross comparative approach ensured themes supported by data
Discussion to reach consensus concerning theme development

Interpretation of findings
Exploration of relationships between themes/ interpretation of significance
Refinement of analysis through search for ‘deviant’ cases that appeared to contradict emergent findings

Illustration of theme development: Perceived positive and negative aspects of orthotic devices (Theme 3)

Examples of codes identified in the transcripts:
-Positive aspects (relating to independent mobility):
‘transformative’; *‘it changed my life’; ‘they give me independence’*

-Negative aspects (relating to poor fit/ inadequate support):
‘it doesn’t stay on my knee as I am walking’; *‘there’s not benefit in it’*; *‘it rubs your skin, I can’t wear it’*

Codes merged under unifying ‘umbrella’ theme: Perceived positive and negative aspects of orthotic devices

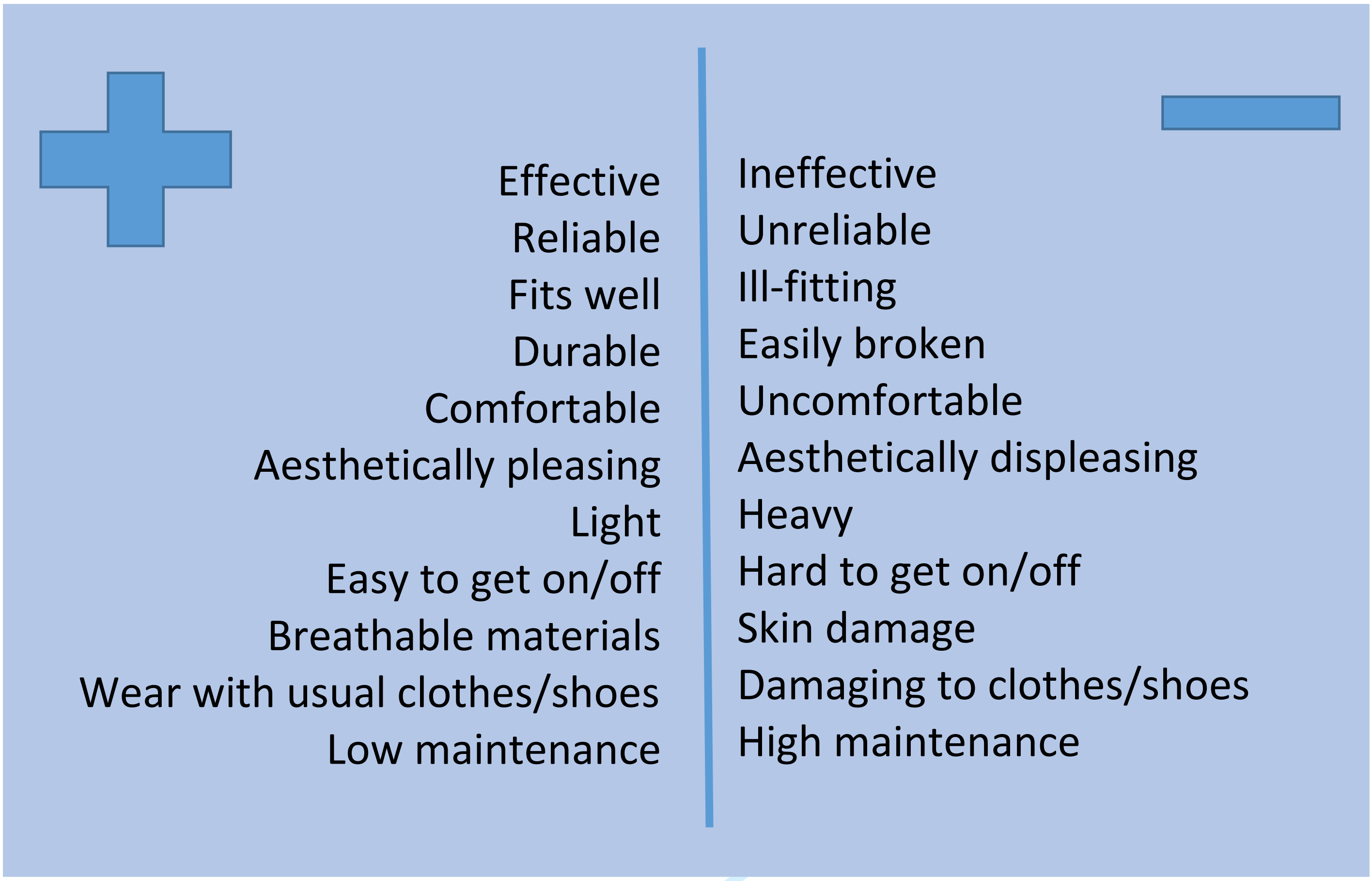

Figure 2 Study participants' perceptions of positive and negative aspects of their orthoses

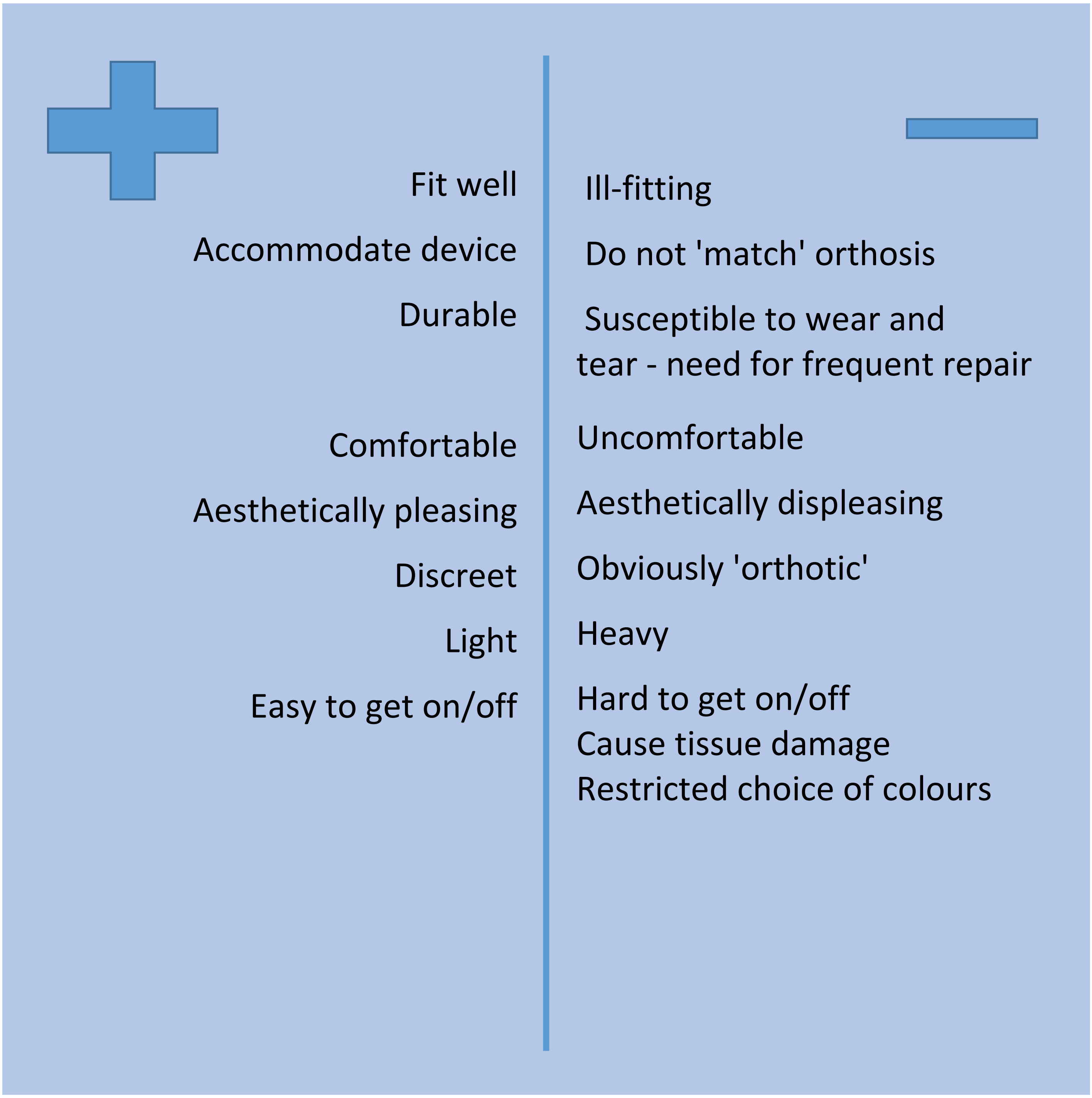

Figure 3 Study participants' perceptions of positive and negative aspects of footwear

Appendix 1 Interview Topic Guide

Researcher to ask brief details about age, occupation, ethnicity, family members, etc. to frame and contextualise the interview

-Can you tell me a little bit about yourself and how you came to have your orthotic device(s) (such as brace or callipers)? [explore important aspects further, such as diagnosis, referral, care pathway, length of time having an orthosis]

-How has the orthotic device impacted on your daily life?

-[from answer to above question] – What factors have affected you most and why? [explore important aspects further]

-What do you like, or not like, about your orthotic device? Which factors influence your decisions to use your orthotic device and why are they important?

-How effective do you think your device is in the management of knee instability?

-How do you feel about the treatment you have received in connection with your knee instability?

-How could this have been improved?

-What are the goals of treatment that matter most to you?

-How do you feel about the interactions you have had with HCPs? How could these have been improved?

-Any other aspects that have not already been discussed or you would like to expand upon?

For peer review only

COREQ (COnsolidated criteria for REporting Qualitative research) Checklist

A checklist of items that should be included in reports of qualitative research. You must report the page number in your manuscript where you consider each of the items listed in this checklist. If you have not included this information, either revise your manuscript accordingly before submitting or note N/A.

Topic	Item No.	Guide Questions/ Description Reported on	Page No.	Explanation
**Domain 1: Research team and reflexivity**				
**Personal characteristics**				
Interviewer/facilitator	1	Which author/s conducted the interview or focus group?	4	Dorothy McCaughan (DM) and Cath Jackson (CJ)
Credentials	2	What were the researcher's credentials? E.g. PhD, MD	n/a	(DM) MSc; BA<br>(CJ) PhD
Occupation	3	What was their occupation at the time of the study?	n/a	(DM) Research Fellow<br>(CJ) Senior Research Fellow
Gender	4	Was the researcher male or female?	n/a	Female
Experience and training	5	What experience or training did the researcher have?	4	(DM) Registered General Nurse; MSc (Nursing); BA (English)<br>(CJ) PhD; MSc; BA<br>Both researchers have previously undertaken qualitative research and have published extensively.
**Relationship with participants**				
Relationship established	6	Was a relationship established prior to study commencement?	n/a	No, participants had no relationship with the interviewer(s) prior to the study.
Participant knowledge of the interviewer	7	What did the participants know about the researcher? e.g. personal goals, reasons for doing the research?	n/a	Participants had no knowledge of the interviewer(s) prior to the study. Prior to participation in the study, participants received a patient information sheet which included details of the study and brief background information concerning the interviewer(s).
Interviewer characteristics	8	What characteristics were reported about the interviewer/ facilitator? e.g. Bias, assumptions, reasons and interests in the research topic	4	Relevant details reported in the manuscript. Data were collected by DM and CJ, who jointly analysed the data. DM and CJ each have many years' experience of applied health services research.
**Domain 2: Study design**				
**Theoretical framework**				
Methodological orientation and theory	9	What methodological orientation was stated to underpin the study? e.g. grounded theory, discourse	4	We conducted a thematic analysis of the data using 'Framework' method,

For peer review only

		analysis, ethnography, phenomenology, content analysis		underpinned by an inductive, cross-comparative approach.
**Participant selection**				
Sampling	10	How were participants selected? e.g. purposive, convenience, consecutive, snowball	4	Purposive sampling was used to identify participants across a range of conditions, age, gender, length of time fitted with an orthosis, and regions across England.
Method of approach	11	How were participants approached? e.g. face-to-face, telephone, mail, email	4	Clinical staff approached 19 patients attending NHS clinics; five people were recruited through contacts with charities/patient support groups.
Sample size	12	How many participants were in the study?	4	24 people took part in an interview for the study.
Non-participation	13	How many people refused to participate or dropped out? Reasons?	n/a	Two people were disinclined to take part in the study (due to comorbidities); no participant dropped out of the study.
**Setting**				
Setting of data collection	14	Where was the data collected? e.g. home, clinic, workplace	4	Data were collected in the homes of 21 participants; three people preferred to be interviewed by telephone.
Presence of nonparticipants	15	Was anyone else present besides the participants and researchers?	4	In two instances, the spouses/partners of participants were present and contributed to the interview.
Description of sample	16	What are the important characteristics of the sample? e.g. demographic data, date	4	Participants were adults diagnosed with a neuromuscular or CNS condition who had been offered an orthotic device for knee instability. Participant diagnoses included poliomyelitis, multiple sclerosis, Charcot-Marie-Tooth disease, spinal injury, stroke and spina bifida.
**Data collection**				
Interview guide	17	Were questions, prompts, guides provided by the authors? Was it pilot tested?	Appendix 1	A topic guide was used. This evolved as the study progressed. This is shown in Appendix 1.
Repeat interviews	18	Were repeat interviews carried out? If yes, how many?	n/a	No, repeat interviews were not conducted.
Audio/visual recording	19	Did the research use audio or visual recording to collect the data?	4	Audio recording was used to collect data.
Field notes	20	Were field notes made during and/or after the interview or focus group?	n/a	Field notes were made after the interviews, when required.

For peer review only

Duration	21	What was the duration of the interviews or focus group?	4	It varied between participants, from 45-90 minutes, with an average of about 1 hour.
aData saturation	22	Was data saturation discussed?	4	Yes, we state that data collection continued until no new information was forthcoming.
Transcripts returned	23	Were transcripts returned to participants for comment and/or correction?	n/a	No, this was not requested by any of the participants and audiotaping was clear.
**Domain 3: analysis and findings**				
**Data analysis**				
Number of data coders	24	How many data coders coded the data?	4	DM and CJ coded the data.
Description of the coding tree	25	Did authors provide a description of the coding tree?	Figure 1	The coding tree reflected the topic guide. Figure 1 provides a visual illustration of the coding and analysis of data.
Derivation of themes	26	Were themes identified in advance or derived from the data?	4/5	Themes were derived from the data, not in advance, although the topic guide provided a framework for data collection.
Software	27	What software, if applicable, was used to manage the data?	4	Data were managed using NVivo software.
Participant checking	28	Did participants provide feedback on the findings?	n/a	All participants were provided with a summary of the study findings.
**Reporting**				
Quotations presented	29	-Were participant quotations presented to illustrate the themes/findings?<br>-Was each quotation identified? e.g. participant number	5-12	Yes quotations are presented, along with the anonymised participant ID number.
Data and findings consistent	30	Was there consistency between the data presented and the findings?	5-12	Yes, we aimed to provide clarity between the data presented and the findings. Statements in text are supported by relevant quotations.
Clarity of major themes	31	Were major themes clearly presented in the findings?	5	Major themes were clearly presented with separate headings. Theme headings were summarised in Figure 2.
Clarity of minor themes	32	Is there a description of diverse cases or discussion of minor themes?	5-12	Whilst highlighting the main themes, we also incorporated a range of responses and drew attention to atypical/negative cases.

Developed from: Tong A, Sainsbury P, Craig J. Consolidated criteria for reporting qualitative research (COREQ): a 32-item checklist for interviews and focus groups. International Journal for Quality in Health Care. 2007. Volume 19, Number 6: pp. 349 – 357. Once you have completed this checklist,

For peer review only

please save a copy and upload it as part of your submission. DO NOT include this checklist as part of the main manuscript document. It must be uploaded as a separate file.

For peer review only

Orthotic management of instability of the knee related to neuromuscular and central nervous system disorders: a qualitative interview study of patient perspectives

Journal:	*BMJ Open*
Manuscript ID	bmjopen-2019-029313.R1
Article Type:	Original research
Date Submitted by the Author:	09-Aug-2019
Complete List of Authors:	McCaughan, Dorothy; University of York, Health Sciences<br>Booth, Alison; University of York, UK, Department of Health Sciences<br>Jackson, Cath; Valid Research Ltd<br>Lalor, Simon ; Queen Mary's Hospital, St George's University Hospitals NHS Foundation Trust,, Orthotics ; Royal Children's Hospital Melbourne, Orthotics/prosthetics<br>Ramdharry, Gita; Queen Square Centre for Neuromuscular Diseases<br>O'Connor, Rory J.; Univ Leeds<br>Phillips, Margaret; Derby Hospitals/ Nottingham University, Rehabilitation<br>Bowers, Roy; University of Strathclyde, Glasgow, UK, Department of Biomedical Engineering<br>McDaid, Catriona; University of York, York Trials Unit
**Primary Subject Heading**:	Health services research
Secondary Subject Heading:	Qualitative research, Rehabilitation medicine
Keywords:	orthoses, knee instability, patient perspectives, qualitative

Manuscript

Orthotic management of instability of the knee related to neuromuscular and central nervous system disorders: a qualitative interview study of patient perspectives

Authors

Dorothy McCaughan[1], Alison Booth[1], Cath Jackson[2], Simon Lalor[3], Gita Ramdharry[4], Rory J O'Connor[5], Margaret Phillips[6], Roy Bowers[7], Catriona McDaid[1]

Institutional addresses

1. Department of Health Sciences, University of York, York, UK
2. Valid Research Ltd., Sandown House, Sandbeck Way, Wetherby, UK
3. Queen Mary's Hospital, St George's University Hospitals NHS Foundation Trust, London, UK
4. Queen Square MRC Centre for Neuromuscular Diseases, National Hospital for Neurology and Neurosurgery & UCL Institute of Neurology, London, UK
5. NIHR Leeds Biomedical Research Centre, Leeds Teaching Hospitals NHS Trust, Chapel Allerton Hospital, Leeds, UK
6. Royal Derby Hospital, Derby, UK
7. Department of Biomedical Engineering, University of Strathclyde, Glasgow, UK

Corresponding author:
Dorothy McCaughan, Department of Health Sciences, University of York, York YO10 5DD, UK
Email: dorothy.mccaughan@york.ac.uk

ORCID iD https://orcid.org/0000-0001-5388-2455

For peer review only - http://bmjopen.bmj.com/site/about/guidelines.xhtml

ABSTRACT

Objectives Adults with knee instability related to neuromuscular disorders or central nervous conditions often experience mobility problems and rely on orthoses to improve function and mobility. Patient views of device effectiveness and acceptability are under-explored. Our study aimed to elicit device users' perspectives regarding fitting, acceptability, effectiveness and use of orthoses, and identify important treatment outcomes.

Design Qualitative descriptive study using in-depth semi-structured interviews. Interview transcriptions were coded and thematically analysed, using 'Framework'.

Setting and Participants A purposive sample of 24 adult users of orthotic devices. Nineteen patients were recruited across three National Health Service (NHS) sites, and five people through charities/patient support groups in England. Half of the participants had been diagnosed with poliomyelitis, and the remainder with multiple sclerosis, Charcot–Marie–Tooth (CMT) disease, spinal injury or spina bifida, and stroke. The median age of participants was 64.5 years (range 36–80 years).

Results Patients' medical condition impacted significantly on daily life. Participants relied on orthotic devices to enable engagement in daily activities. Patient goals for mobility were linked to individual circumstances. Desired treatment outcomes included reduction in pain, trips and falls, with improved balance and stability. Effectiveness, reliability, comfort and durability were the most valued features of orthoses and associated with reported use. Obtaining suitable footwear alongside orthotic devices was a significant concern. Time pressures during device fitting were viewed negatively.

Conclusions Orthotic devices for knee instability play a crucial role in promoting, maintaining and enhancing physical and psychological health and well-being, enabling patients to work, engage in family life and enjoy social activities. Future research should consider how best to measure the impact of orthotic devices on patient quality of life and daily functioning outside the clinic setting, as well as device use and any adverse effects.

This qualitative study was retrospectively registered as Current Controlled Trials ISRCTN65240228.

Key words: knee instability, neuromuscular conditions, central nervous system conditions, orthoses, patient perspectives, qualitative study

Word count: 5,206 (without quotations); 6,314 (including quotations)

For peer review only - http://bmjopen.bmj.com/site/about/guidelines.xhtml

ARTICLE SUMMARY

Strengths and limitations of this study

- This is the first study specifically designed to explore the views and experiences of patients regarding effectiveness and acceptability of orthotic devices, and to identify outcomes that are important for people who have been fitted with an orthotic device for knee instability, across a broad range of conditions.
- Our study captured rich data concerning issues identified as significant by participants, providing important new insights.
- A full range of neuromuscular and central nervous system conditions (for example, myasthenia gravis and muscular dystrophy) were not represented in the sample.
- Nonetheless, the study included people with regular and sustained contact with orthotic services in connection with knee instability.

INTRODUCTION Adults with knee instability related to neuromuscular disorders (NMD) or central nervous system (CNS) conditions may experience pain, loss of balance, falls and mobility problems due to primary impairments of muscle weakness and/or sensory impairment. Muscle weakness can lead to instability of the joints which can be particularly problematic in the lower limbs during weight bearing tasks; if the muscles cannot generate sufficient force to resist gravity, then the lower limb joints can be unstable or collapse. In addition, if sensory feedback is also impaired, people may be unaware of the position of their joints or if they have moved, so increasing the risk of instability.

Prescription of devices to stabilise the knee will depend on which muscles are weak that act on the knee joint. KAFOs are more often prescribed when proximal lower limb weakness contributes to knee instability, e.g. weakness of the quadriceps. The KAFO will stabilise the position of the femur on the tibia and the tibia over the foot, thus stabilising the knee. If lower forces are required and the ankle is stable, knee braces can be used to stabilise the femur on the tibia to support the knee. In the case of focal distal weakness, e.g. the plantar flexal muscles, AFOs can stabilise the ankle during stance, so maintaining the position of the tibia against gravity and preventing a collapse of the knee into excessive flexion or hyper-extension.

Orthotic devices can offer support, align or correct deformities, or improve function.[1,2] A knee–ankle–foot orthosis (KAFO) can be prescribed when other forms of bracing, such as an ankle–foot orthosis (AFO) or knee orthosis (KO), are insufficient to adequately control knee instability, due to weakness or muscle imbalance. However, usage can be low.[3-5] Accurate figures relating to the number of people treated with orthotic devices are lacking, reflecting the challenges associated with obtaining data on orthotic services in England, partly due to the complexity of pathways of care[6,7]

Literature concerning the views and experiences of users of lower limb orthotic devices for knee instability is scant.[8] A review of 10 studies [9] examined patient use of

For peer review only - http://bmjopen.bmj.com/site/about/guidelines.xhtml

lower-limb orthotic devices (e.g. knee brace and AFOs) and orthopaedic shoes, but none of the included studies related to use of KAFOs. The authors reported a wide range of rates of non-use, mainly due to pain, discomfort and non-cosmesis. A recent survey of orthoses users in Belgium[2] similarly reports varying levels of satisfaction with devices. While inclusion of 'open' questions in the survey elicited 'free text' responses from participants, the underlying reasons for device satisfaction and use were not explored in depth, as this requires the use of appropriate qualitative methods. Results from focus group studies have appeared recently, but do not elucidate KAFO-users' perspectives. Swinnen et al.[10] conducted 4 focus groups with 20 patients diagnosed with multiple sclerosis that revealed that stigmatization, difficulties in putting on their lower limb orthosis (AFO) and aesthetic aspects were implicated in non-use. A focus group with eight AFO users living in the Netherlands to gain insight into importance of device related activities to AFO-users found that participants ranked walking as the most important of 11 different activities.[11] However, this latter small-scale study did not include any KAFO users, whose priorities for treatment outcomes may differ.

Understanding of the treatment outcome priorities of people with knee instability related to neuromuscular and CNS conditions is limited, and the range of different outcome measures used to evaluate patient outcomes following device fittings [12,13] makes it difficult to draw conclusions about effectiveness as data cannot be pooled for meta-analysis. Our study aimed to: (i) explore patient perceptions regarding fitting/acquisition, acceptability, effectiveness and usage of orthoses, and (ii) identify the outcomes that are important for people who have been fitted with an orthotic device for knee instability, across a broad range of conditions. The qualitative study reported here comprises one strand of a larger study.[8] The report here focuses on treatment goals and outcomes of importance to patients, and device acceptability, contextualised within individuals' experiences of their specific medical condition. Further details of patients' views and experiences of orthotic service delivery can be found in the report of the overall study, which includes results from a systematic literature review, a survey of health-care professionals, and a costing analysis of knee-ankle-foot orthoses.[8]

METHODS

Study design

Semi-structured individual interviews were undertaken (during 2014-2015) with adult users of orthotic devices. Qualitative approaches are well-suited to investigation of phenomena about which little is known, while semi-structured interviews offer flexibility in data collection and generate rich narratives, allowing the researcher to analyse how participants make sense of the topic under investigation.[14] We followed the Consolidated Criteria for Reporting Qualitative Studies guidelines for reporting qualitative research.[15]

Patient and public involvement

The study was designed in collaboration with a patient advisor, herself a user of an orthotic device, who contributed to the design and piloting of the interview topic guide (Appendix 1).

Sampling

For peer review only - http://bmjopen.bmj.com/site/about/guidelines.xhtml

Inclusion criteria for the study were adults (≥ 16 years for NHS participants; > 18 years of age for non-NHS participants) with a neuromuscular disorder who have impaired walking ability primarily due to instability of the knee. Neuromuscular disorder included conditions that primarily affect the peripheral nerve, muscle and neuromuscular junction, for example motor neurone disease, muscular dystrophy, myasthenia gravis, spinal muscular atrophy, CMT disease, poliomyelitis, myopathies and inclusion body myositis. People with knee instability that was related to CNS conditions were also included, for example spinal cord injury, spina bifida and stroke. Participants were people who were able to give informed consent.

Participants were purposively sampled [16] to reflect a range of conditions, ages, gender, length of time fitted with an orthosis, and regions across England. Potential participants who met the study inclusion criteria were approached by clinicians who were known to them, in three orthotic service/medical rehabilitation NHS outpatient settings, one located in the north, one in the south and one in the middle of England. Patients were informed about the study by the clinicians; those who expressed an interest were given written information about the study, including the researchers' contact details if they wished to learn more and/or discuss taking part in a qualitative interview. Additionally, study information was provided to the chairpersons/lead representatives of the British Polio Fellowship, Charcot-Marie-Tooth UK, the FSH (facioscapulohumeral muscular dystrophy) Support Group UK and the Muscular Dystrophy Campaign, with a request to forward this to their members, who were invited to contact the qualitative researcher directly. Twenty-four people participated, nineteen recruited through NHS clinics and five through charities/patient support groups.

Participants were adults diagnosed with a neuromuscular or CNS condition who had been offered an orthotic device for knee instability of varying degrees of severity, that negatively affected their gait. Twelve had been diagnosed with poliomyelitis, and the remainder with multiple sclerosis, Charcot–Marie–Tooth (CMT) disease, spinal injury, stroke and spina bifida. Participants' ages ranged from 36 to 80 years (median 64.5 years). Half worked part or full-time while the remainder were retired; most (18) lived with their spouse and/or other family members. Further details relating to duration and severity of patients' conditions, and reported device use, can be found in Appendix 2: Participant Characteristics.

Ethics approval was gained for the study to take place and written consent was obtained from all patients who took part in an interview.

Data collection

A topic guide was developed, based on the aims of the research to explore patient perceptions of using an orthosis and goals for treatment; we sought to develop an instrument sufficiently structured to ensure consistency in information gathering, but flexible enough to allow participants to recount their individual experiences.[17] Input from the patient advisor helped guide relevance of content and comprehensiveness of the topic guide. Two experienced qualitative researchers (DM and CJ) undertook the interviews. Twenty-one interviews were conducted in participants' homes; three

For peer review only - http://bmjopen.bmj.com/site/about/guidelines.xhtml

people requested a telephone interview as this was more convenient. In two cases, family members were present during the interview and their comments were included in data analysis. Interviews lasted approximately one hour and all were audio-recorded. Data collection continued until no further pertinent information was forthcoming (after 24 interviews). Audio-recordings were transcribed verbatim by an experienced transcriber, and a random sample of six transcripts checked for accuracy by DM and CJ.

Data analysis

Data were analysed for thematic content using the 'Framework' method [18] (see figure 1). Framework is a flexible tool, not aligned with a particular epistemological, philosophical, or theoretical stance, but adaptable to various qualitative approaches aiming to generate themes. Framework approach incorporates 'charting' or tabulation of data, which involves summarising and re-arranging data according to thematic content, enabling the analyst to easily see the range of data across cases and under themes.[19] NVivo software package [20] facilitated data handling and retrieval and enabled comparison within and between cases. Analysis was systematic and iterative [19] and involved close reading and detailed coding of transcripts. Guided by the research questions, our approach to data analysis balanced both inductive and deductive orientations, as we sought to transfer the 'raw' data into a new and coherent description of the phenomena under scrutiny. [21] To promote analytical rigour, coding was cross-checked in four early transcripts by the two experienced qualitative researchers (DM, CJ) involved in the study. We sought to identify commonalities and differences in the data, with the aim of drawing descriptive and/or explanatory conclusions clustered around themes.[17] Differences in identification and development of themes were discussed until consensus was reached, and the thematic framework accordingly modified and expanded to accommodate all participant responses across the data set. Examination of negative or 'deviant' cases [22] helped to develop and refine the analysis.

RESULTS

Five principal themes were identified: (1) impact of NMD and CNS conditions on walking and mobility; (2) fitting/acquisition and use/wearing of orthotic devices; (3) perceived positive and negative aspects of orthotic devices; (4) footwear; (5) desired treatment goals and outcomes. These themes are described below. Details relating to further themes/subthemes can be found in the main report.[8]

1. Impact of NMD and CNS conditions

Participants' mobility was impaired by a range of symptoms and sequelae (see Box 1) associated with their condition, including pain, muscle weakness, fatigue, loss of balance, unsteady gait, and frequent trips and falls.

For peer review only - http://bmjopen.bmj.com/site/about/guidelines.xhtml

Box 1 Symptoms and sequelae associated with NMD and CNS conditions

Reported symptoms
-Pain (sometimes severe) in knee or ankle joints
-Hyperextension of knee joint
-Muscle weakness in the lower limb and/or of the muscles supporting joints
-Limbs of unequal length
-Feet ‘frozen’ in abnormal position
-Varying degrees of paralysis in lower limbs and feet
-Toes that ‘curl inwards’
-Drop foot
-Fatigue

Reported sequelae
-Frequent falls, often preceded by feeling that leg is about to ‘give way’ under them
-Loss of sense of balance and stability
-Lop-sided gait
-‘Dragging’ of feet
-Frequent trips, especially on uneven surfaces
-Difficulty standing for long or walking any but short distances
-‘Wear and tear’ in unaffected or ‘good’ limb due to transfer of weight and effort in walking

Limited mobility was associated with feelings of fearfulness and anxiety, resulting in diminished self-confidence and independence. People in employment were particularly anxious to gain or retain mobility as far as their condition allowed. Many participants described being proactive in trying to postpone or abate the impact of future deterioration on independent mobility, by keeping their weight under control, exercising regularly, and undertaking exercise programmes recommended by physiotherapists. Reduced mobility was said to limit the pursuit of enjoyable activities such as gardening and walking, and could lead to social isolation. Retaining the ability to drive was viewed as important for both work and leisure purposes.

‘The last time I was on the tube I got pushed…and I lack confidence and get scared, so if I haven’t got somebody with me, I won’t use the tube.’ (Participant (P) 23)

‘I was in a very well paid job… and I saw how much I deteriorated in the last 5 years. . . I try to help myself…like with my weight… I have to work on that and my exercise . . . I am active.’ (P22)

‘Obviously I walk slower, so I am on my own quite a bit because I have got good friends who will walk with me, but a lot of people [won’t] . . . so it does actually make you an alone sort of person’ (P2)

‘I’ve got an automatic car . . . I’ve got a mobility car with a hoist in the back which can pick up my scooter.’ (P18)

Unsurprisingly, obtaining the ‘right’ orthotic device(s) to help with current and future mobility emerged as a central concern of all study participants.

2. Fitting/acquisition and use/wearing of orthotic devices

For peer review only - http://bmjopen.bmj.com/site/about/guidelines.xhtml

Fitting/acquisition of knee–ankle–foot orthoses (KAFOs)

Participants who used a KAFO (n=12) all had a diagnosis of poliomyelitis. These participants emphasised the importance of having a 'spare' KAFO for use during the period of being fitted for, and 'breaking in', a new device. Fitting was frequently described as a slow process, involving numerous appointments, often spaced at long intervals, and sometimes cancelled at short notice by the service provider. Attending appointments could mean time away from work, due to lack of 'out of hours' appointments, and some people mentioned difficulties with transportation. The fitting of a new device could be particularly challenging for those with complex condition-related problems; some individuals recalled attending multiple appointments stretching across a 2-3 year period before finally obtaining a device that fully met their needs and was comfortable.

'If you're not getting the appointments, you're not getting the work done' (P19)

'It was not a standard case, it made it much harder and I think for those first 2 or 3 years where it seemed I was going backwards and forwards to [clinic] and I felt that no real progress was being made' (P5)

Many people mentioned the importance of having sufficient time during fitting appointments. 'Double' appointments were said to allow more time for assessment and minor adjustments to devices that promoted optimal fit. Time pressures were perceived as constraining orthotist-patient communication regarding the new device and any adaptations to gait that might be needed. Problems might only come to light after the patient returned home and they could wait some considerable time for another appointment, unable to use the issued orthosis in the interim. Adequate time for full discussion during appointments was highlighted as particularly important to people receiving a KAFO for the first time, who might be struggling to accept and adapt to their orthosis.

'You don't really sort of pick up that sort of a problem when you're having a short appointment…you're only there for 20 minutes, half an hour…you only think about the questions afterwards' (P14)

A minority of participants reported receiving a device so ill-fitting that it was unwearable; participant 15, for example, reported seeking a new KAFO through private (non-NHS) suppliers due to the poor fit of their NHS device.

'It wasn't a question of being uncomfortable, it didn't fit. It just did not fit and they took it away – I didn't even leave with it.' (P8)

'It was too big, too high, so it was going right into my buttocks, it was too wide, so I wasn't getting support at the knee and it was like it wasn't made for me.' (P15)

Participants underscored the importance of being fitted for new footwear (discussed below) at the same time as being fitted for a new KAFO.

'every calliper kind of needs a new boot to kind of fit it, so I was wearing the old calliper and had to wear it with these boots which didn't kind of work with the calliper.' (P23)

Fitting/ acquisition of ankle-foot orthoses (AFO) and knee braces

For peer review only - http://bmjopen.bmj.com/site/about/guidelines.xhtml

Four of the five AFO users were satisfied with the fit of their device. The fifth described her new AFOs as *'too painful to wear'* and causing damage to her skin, and she reverted to using her 'old' devices while waiting for the new ones to be adjusted.

'I've just had a new pair . . . at the moment I've been bedding them in but something is not quite right with them . . . I've worn them for about a month but my foot has started to hurt again and I think the padding is not quite in the right place…' (P21)

Two people (one diagnosed with a spinal injury, one with stroke) had been supplied with 'off the shelf' knee braces through physiotherapy services, but found them ill-fitting and uncomfortable, so that they remained unused.

'I just couldn't bear it. When I walked it just dug in, you can see the bruising….I would have preferred a bespoke one right from the start' (P4)

Use/wearing of orthotic devices

Device usage ranged along a continuum, from full-time use during the day, to little, or no use. Usage was closely linked by participants to capacity for independent mobility and therefore crucial to daily life. Many described their orthosis as integral to their social identity, enabling them to fulfil their desired roles as employee, spouse/partner, parent, and friend.

'I need my surgical appliance to get on with my life . . . it needs to be effective and reliable . . . I want to be in the real world. I love it . . . I'm in a working world and I just want to get on with it' (P2)

Consequently, device malfunction was highly disruptive to 'normal' life.

'if it breaks, you're kind of thrown really . . . I came out of work, it was about half past six, and I got across the road and the calliper just went, it just snapped' (P6)

'As soon as this thing (KAFO) breaks down, I'm in silly street . . . that's not a place I want to go' (P19)

Other people reported using their device depending on the circumstances; for example, using their device outdoors, but not when at home. Intermittent use was also reported during *'breaking in'* of a new device, including alternating between wearing the new and 'old' devices. Reluctance to make the transition from an 'old' to a new device was widespread, due to '*teething troubles'* with the new device, and/or because of the need for gait adjustment, described by participant 18 as *'like having to learn to walk again'.*

Three of the 24 participants reported almost no use of their device as it did not offer adequate support for their knee or ankle. Additionally, a poor fitting device could result in skin damage and/or pain and discomfort, resulting in non-use. The most extreme (and therefore atypical) report of receipt of an ill-fitting orthosis, came from Participant 1 (diagnosed with poliomyelitis in childhood), who commented that she had never worn any of numerous orthoses provided for her, as they had been ineffective, ill-fitting and uncomfortable to wear. During interview, participant 1

For peer review only - http://bmjopen.bmj.com/site/about/guidelines.xhtml

retrieved from elsewhere in the house the KAFO that she had most recently received through orthotic services, still in its plastic wrapper, and described by her as *'a complete waste of NHS money'*. Instead, she preferred to rely on a stick to help her mobilise, while recognising that this was not a good long-term solution, and she said she was finding it increasingly difficult to mobilise.

3. Perceived positive and negative aspects of orthotic devices

Unsurprisingly, participants' perspectives of positive and negative aspects of their orthosis (see figure 2) synchronised with their reports of device use or non-use. Functionality and reliability were overwhelmingly considered more important than cosmesis, though appearance of the device was a significant feature for some of those interviewed (both male and female), who described the *'ideal'* device as light, sleek and discreet.

Aspects of devices viewed positively
Effectiveness of the device to control pain and offer support for the knee or ankle, and/or to assist with lifting the foot, alongside reliability and durability, were the features viewed most positively. Reliability of the device to withstand wear and tear, was highly valued. The people most satisfied with their orthosis referred to the *'transformative'* effects on their lives of a well-fitting and well-functioning device.

'it's [KAFO] *really transformed my life…from here to [place name] may not appear sort of a long distance but for me I don't think I could have done it beforehand, now I can which is a major sort of step forward . . . it's made me much more mobile than I ever was before really'* (P5)

'I was frightened to go out . . . It's just like, they [AFOs] just give me a new lease, you know, they give me my independence to go out on my own…with these splints I've got my independence. I can go out without my husband, I can go out with my friends.' (P21)

Appearance, light-weight, and 'user-friendliness' (the ease with which the device could be donned and doffed, fastenings that are easy to manage and reliable, ease of use with preferred clothing, and 'breathability of the materials used) were secondary concerns, though also appreciated.

'he showed me one at the time, which looked pretty cool . . . it just looked really, really good.' (P8)

'It has carbon fibre, so it's lighter…it's not as wide, I can use most trousers' (P3)

'modern day materials are absolutely fabulous . . . there is no weight in them and they are very strong' (P22)

'It's speedy . . . you can put it on quickly' (P6)

Aspects of devices viewed negatively
Devices providing inadequate knee support, that were poor fitting (resulting in pain, discomfort or skin damage), and/or prone to breakage or malfunction, were described in strongly negative terms.

For peer review only - http://bmjopen.bmj.com/site/about/guidelines.xhtml

'this doesn't stay on my knee, as I am walking it falls right to the bottom. . as I am walking it's slipping down…If it worked, it would be OK . . . there's no benefit in it…it just doesn't do nothing . . . I swear, I feel completely hopeless' (P1)

'we went to [city] and literally we'd just landed at [name] airport and I'm walking to get my suitcase and I fell flat on my face . . . the thingy here, see there, it just went. Really is this screw holding me, I mean can't you think of something stronger than that!… the whole thing is very basic… the holiday was ruined…I couldn't relax… it was a short trip.' (P23)

Breakage or device malfunction represented a crisis, temporarily depriving patients of their independence and interfering with plans and activities. Having a spare device in this situation was important to the majority of patients. They pointed out that even a seemingly simple repair, such as having leather straps replaced when they wear out, could result in difficulties and inconvenience. Other concerns related to discomfort, due to the device being heavy, bulky or cumbersome, devices considered aesthetically displeasing, difficult to put on or take off, and those that caused damage to clothing and/or footwear. Dislike of hook-and-loop fastenings was common; they did not always hold the device securely, could 'snag' on tights, and collect fabric fibres and fluff making them ineffective. Restrictions in choice of clothing and/or footwear to accommodate devices could be irritating; three male participants reported wishing to appear smartly dressed on formal business or social occasions, but having to wear wide-legged trousers to accommodate their device.

'I have buckles, I hate Velcro' (P16)

'it's difficult to get trousers that are really wide . . . so I buy women's jeans . . . from second-hand shops, the charity shops . . . callipers damage them, I can't pay £50–£70' (P16)

Serious skin damage in relation to wearing a KAFO or AFO was rarely reported, though marking or bruising of the skin in connection with newly acquired and/or ill-fitting devices was commonly mentioned, particularly where the knee is in close proximity to the joint on the KAFO. Skin rashes were mainly associated with plastic devices. Examples of 'home grown' measures taken to prevent discomfort and minor skin damage included wearing tights, leggings or pyjamas under the device, adding extra padding at pressure points or, in one case, buying ankle straps that were made of softer leather than those supplied. Warm weather could give rise to skin irritation and several people mentioned looking forward to taking their device off at the end of the warm day to allow their skin to *'breathe'.*

'During the summer I quite often wear pyjama bottoms underneath the straps and leather of the calliper' (P9)

'One year…it was very hot… and I didn't have any tights on… it just rubs your skin, you can't wear it against the skin… I had blisters' (P18)

'It's a bit like taking your shoes off . . . when I take the calliper off the skin can breathe a bit more' (P3)

4. Footwear

For peer review only - http://bmjopen.bmj.com/site/about/guidelines.xhtml

Being fitted for new shoes at the same time as for a new orthosis was considered important and positive and negative aspects of footwear were mentioned by participants (see figure 3). KAFO users diagnosed with poliomyelitis explained they might require each shoe of a pair to differ in width or height as their specific requirements can change over time, and require frequent re-assessment.

'*I had to insist and say, look none of your shoes is fitting me properly . . . I haven't had them reviewed since the 90s and I'm a lot older . . . every time I come, something is wrong.*' (P15)

Delays to the manufacture and delivery of new shoes were experienced as frustrating and disappointing. Shoes were said to require frequent repair as heels and soles can wear out quickly. Participants disliked having to rely on one pair of shoes for everyday use while repairs were being carried out on their other pair; many people said they usually owned only two pairs of orthotic shoes at any one time, regarding two pairs as their maximum entitlement. A good fitting shoe that avoided damage to skin or deeper tissues were priorities. Comfort was generally valued over appearance, although many people expressed a preference for footwear described as *'discreet'*. Heavy or *'clumpy'* orthotic shoes were said to interfere with ease of walking, and to draw attention to their condition. Several participants commented that the grip on the soles of shoes supplied to them was often inadequate for walking on uneven, muddy or icy surfaces, and suggested that a walking-shoe type of sole could improve confidence with walking outdoors.

'there is no point in weeping over kitten heels because I am never going to wear those. But something reasonably presentable, that is not too intrusive, because you've got enough to be getting on in life with without having some people staring at things you might be wearing because obviously I am a bit conscious of the way I do walk . . . and people do stare a bit, so something that can be quite discreet is important.' (P2)

Having a wide choice of colour, fastening (some people found hook-and-loop easier to use than laces), material (leather or suede), and style of shoes, was appreciated, and most people thought that choice and quality of orthotic footwear have improved in recent years. Several people mentioned usually choosing black or brown shoes to '*go with anything'* and as suitable for work, while a few thought these were the only colours available to them. One person insisted on brightly coloured shoes to suit his personality.

'Because of my personality, I like bright shoes, for example, I always wore bright green shoes . . . these are quite bright red really…it is part of your identity.' (P5)

Participants using other types of orthoses (such as a knee brace) reported that they usually purchased shoes from local shops selling footwear, preferring particular brands of 'sturdy' shoes. Other people reported using orthotic devices such as insoles and inserts inside 'normal' shoes, either supplied through orthotic services or bought from commercial suppliers.

5. Desired treatment goals and outcomes

Effective support for the affected (knee or ankle) joint with concomitant reduction of joint pain was the prime goal for most participants. Individuals wished to be as confidently mobile as their condition would allow, achieved through reduced anxiety

For peer review only - http://bmjopen.bmj.com/site/about/guidelines.xhtml

that their joint might suddenly *'give way',* and avoiding trips and falls. Preventing future deterioration of mobility (as far as feasible in relation to their specific condition) was a significant long-term goal. It was clear from participants' accounts that their desired treatment goals related to both physical and psychosocial aspects of their everyday lives. Cited benefits of independent and safe mobility included increased employment opportunities, ability to participate in day-to-day family activities, and social events with friends and colleagues, as well as independent travel, and regular exercise, all regarded as vital to current and future physical and mental well-being.

'I just do not want to become housebound, so that primarily is the objective of my orthotics.' (P22)

'I need my surgical appliance to get on with my life . . . it needs to be effective and reliable' (P2)

'It's just enabling me to keep going as long as possible which is really important to me.' (P10)

Most study participants seemed to have accepted that they were unlikely to ever be able to walk very fast, or for very far, without some degree of fatigue, and they showed little interest in increasing the speed of their walking, or distance covered, as treatment goals per se. Instead, goals were expressed in terms of enablement of mobility, within the context and confines of individual circumstances, such as being able to work full-time and undertake world-wide travel, walk a few hundred metres to nearby shops, or being able to take just a few steps.

'My walking is so limited. If I can stand and get something out of a cupboard and walk a few steps and get back to the wheelchair or whatever, that's what I can do, and that's what I need to do. For example in [name] mother's house I can't get the wheelchair in . . . because of the threshold, so I have to get out, walk a couple of steps over the threshold and then get back in the wheelchair and to be able to just walk those few steps to stand is an enormous advantage.' (P16)

DISCUSSION

Principal findings

Participants described knee instability as compromising their ability to pursue desired daily activities and as having a negative impact on their social life. Effectiveness, reliability, comfort and durability were the most valued features of orthoses, and seemingly closely linked to their use, while cosmesis was perceived as of secondary importance. Reduction in pain, trips and falls, and improved balance and stability, promoting independent mobility were the most important outcomes to participants. The standard used by many participants to assess the effectiveness of their device was the extent to which it enabled them to engage in a broad range of activities viewed as important for physical and mental well-being. For some, even limited independent movement was regarded as a valued outcome. Comfort (fit and weight) of shoes, appearance, and availability of choice regarding colour, materials, types of fastenings and style were all considered important by participants in our study, factors that seemed to be associated with likely wearing of prescribed shoes. Male participants were as equally concerned as females with cosmesis of devices and shoes. Participants highlighted various issues relating to the fitting/acquisition of devices, such as long waits between appointments, and time pressures during appointments, that resulted in reduced opportunities for contact and communication with clinicians. As a result, the period over which fitting took place might be

For peer review only - http://bmjopen.bmj.com/site/about/guidelines.xhtml

prolonged, and patients were sometimes left feeling they did not receive the support and education they required to optimise adaptation to their device, especially where the device was a KAFO.

Results in the context of other studies

As part of the same project we also undertook a systematic review of the evidence on effectiveness of orthotic devices for the management of knee instability in adults with a neuromuscular or CNS disorder.[23] The review found that effectiveness studies focused on outcomes such as gait quality and energy consumption, assessed in the clinic setting. While important outcomes, this focus neglects many outcomes identified as important by participants in this qualitative study.

Our findings align with Schaffalitzky et al.'s [24] study with 24 lower limb prosthetic users that revealed that even small gains in mobility provided by a device may constitute a successful outcome from the patient's perspective. Evidence relating to acceptability and use of KAFOs is limited. Swinnen et al.[2] have reported device functionality and comfort as more highly valued than appearance amongst 33 patients with neurological conditions (28 of whom used an AFO), views also widely held by participants in our study. Our study findings also echo results from previous studies of patients' views of AFOs [25-27] indicating that orthoses that were ill-fitting, considered uncomfortable, heavy, cumbersome and unsightly, and which drew attention to disability, were less acceptable to patients.

The important contribution of footwear to people's identity and self-expression is well recognised [28,29] and was a significant concern for both male and female participants in our study, although good fit and comfort were prioritised.

Participants in our study repeatedly emphasised the need for individually tailored care, indicating the need for a service model such as the one advocated by Hutton and Hurry,[30] where orthotic products are not viewed as commodities, but as individually prescribed solutions suited to each patient's personal needs. Lengthy waiting times to access orthotic services and frequent delays in the provision and repair of custom-made KAFOs and footwear were commonly reported in our study, as elsewhere[7, 31, 32] as impeding device use. Our study participants also emphasised the need for intensive support when receiving a device for the first time, as they adjust to altered self-image. They suggested that in addition to technical information, patients may require psychological support, which has been highlighted previously.[3, 30, 33]

Strengths and weaknesses of the study

Our study used robust methods, including development of the topic guide with a patient and clinicians, and quality assurance of data collection, coding and thematic analysis. The role of the patient advisor in interpretation of the study findings was, however, limited, due to unforeseen circumstances. A full range of neuromuscular and CNS conditions (for example, myasthenia gravis and muscular dystrophy) are not represented in the sample, which is a limitation. Nonetheless, the study included people with regular and sustained contact with orthotic services in connection with knee instability. In addition, we recruited sufficient patients to achieve data saturation,[34] within the limits recommended by Hennink et al.[35] to achieve *'meaning saturation'*, which enables a richly textured understanding of issues. Inclusion of

For peer review only - http://bmjopen.bmj.com/site/about/guidelines.xhtml

different age groups revealed some differences in perspectives. Whereas younger participants in our study emphasised the importance of their orthosis in enabling them to undertake full-time employment and function in the role of family breadwinner, older (often retired) people often talked about device use in facilitating their involvement in family and leisure activities that helped them feel connected to the physical and social world beyond their home. We recognise that the views of our study participants may not be reflective of the broader population of people with knee instability due to NMD and CNS conditions; for example, only one person with a diagnosis of stroke was included, and patients younger than 36 years old were absent from the study, and their perspectives warrant investigation. Nor did we set out to systematically assess knee instability in individual patients, using current assessment techniques and grading scales, which may be viewed as a limitation. Rather, we relied on patients to report knee instability as they experienced it, and its implications for daily life. Owing to the comparatively under-investigated nature of our research topic, transferability of our study findings may be less salient than 'sensitising' readers to new information,[19] captured through in-depth interviews, promoting new ways of thinking about patients' perspectives of using orthotic devices.

Applicability to clinical practice and suggestions for further research
The findings have implications for the training of healthcare professionals involved in the prescribing, fitting and follow-up of patients using orthoses to control knee instability who have complex neuromuscular or central nervous system conditions. To maximise patient acceptance and use of prescribed devices, it is important that the relevant healthcare professionals (orthotists, podiatrists, physiotherapists, occupational therapists) have the appropriate skills, and the necessary time during consultations, to establish a relationship where patients feel listened to, and that supports patients to identify acceptable management strategies and achieve desired treatment outcomes. Patient education and self-efficacy could be enhanced through more flexible routes of referral to orthotic services, including patient self-referral; having a 'named' orthotist as case manager for an individual patient's care, to improve continuity of care; and closely integrated multidisciplinary team working that includes opportunities for shared learning in communications skills. [7, 36] More research is needed to assess the impact of such initiatives.

Development and use of a core set of patient-reported outcome measures in the clinical and research setting would facilitate assessment of the effectiveness of different devices and management strategies. Brehm et al.[37] have suggested development of a core set of outcomes for studies of lower limb orthoses, based on the World Health Organization International Classification of Functioning Disability and Health.[38] To date, however, there has been little qualitative research to 'unpack' patient perspectives of terms such as 'satisfaction' and 'effectiveness', although these constructs have been incorporated into existing outcome measures.[13] We hope that our findings will contribute to the development of outcome measures that reflect patients' priorities.

Our research highlights important aspects that should be included from the patient experience, such as quality of life and psychological well-being, as reflected through ability to engage in a broad range of activities viewed as important for physical and

For peer review only - http://bmjopen.bmj.com/site/about/guidelines.xhtml

mental well-being, and reduction in pain, trips and falls. It is important that patients are involved in any future work to develop a core outcome set.

CONCLUSION

Orthotic devices for knee instability play a crucial role in promoting, maintaining and enhancing physical and psychological health and well-being, enabling patients to work, engage in family life and enjoy social activities. Reduction in pain, trips and falls, and improved balance and stability, linked to potential for independent mobility, were regarded as important treatment outcomes. Time constraints and delays in orthotic service delivery can adversely affect timely provision, and patient use, of orthotic devices. Future research should consider how best to measure the impact of orthotic devices on patient quality of life and daily functioning outside the clinic setting, as well as device use and any adverse effects.

Author affiliations

1. Department of Health Sciences, University of York, York, UK
2. Valid Research Ltd., Sandown House, Sandbeck Way, Wetherby, UK
3. Queen Mary's Hospital, St George's University Hospitals NHS Foundation Trust, London, UK
4. Queen Square MRC Centre for Neuromuscular Diseases, National Hospital for Neurology and Neurosurgery & UCL Institute of Neurology, London, UK
5. NIHR Leeds Biomedical Research Centre, Leeds Teaching Hospitals NHS Trust, Chapel Allerton Hospital, Leeds, UK
6. Royal Derby Hospital, Derby, UK
7. Department of Biomedical Engineering, University of Strathclyde, Glasgow, UK

Acknowledgements We gratefully acknowledge all the participants who took part in interviews. We would especially like to thank our patient advisor, Ms Sam Bunting, in her role as patient representative. We also wish to acknowledge the contribution of Lisa Dyson to conception of the study.

Author Contributions CMcD, AB, RB, GR, SL and MP designed the study. RJOC provided clinical advice. DM and CJ recruited participants and conducted interviews. Transcripts were coded and analysed by DM with input from CJ. DM wrote the first draft of the manuscript, which was revised with CMcD and AB. All authors commented on and approved the final manuscript.

Funding This project was funded by the National Institute for Health Research (NIHR) HTA Programme (project number 13/30/02) and has been published in full in Health Technol Assess 2016;20(55). Further information available at https://www. journalslibrary. nihr. ac. uk/ programmes/ hta/ 133002/#/ This report presents independent research commissioned by the NIHR. The views and opinions expressed by authors in this publication are those of the authors and do not necessarily reflect those of the NHS, the NIHR, MRC, CCF, NETSCC, the HTA programme or the Department of Health.

For peer review only - http://bmjopen.bmj.com/site/about/guidelines.xhtml

Competing interests During this study, SL was an employee of Opcare, a company that provides orthotic and prosthetic services to the UK NHS. This company does not manufacture orthotic devices, although a sister company ORTHO C FAB does.

Patient consent for publication Not required.

Ethics approval The study received research ethics (REC reference 14/LO/1132) and governance approvals to recruit patients through NHS sites. The University of York's Department of Health Sciences' Research Governance Committee gave approval to recruit study participants via patient support groups/charities. Written consent was obtained from all patients who took part in an interview; relatives gave verbal consent for anything they said to be included in any publications. All participants were given assurances concerning confidentiality and anonymity and informed that they could withdraw from the study at any time.

Provenance and peer review Not commissioned; externally peer reviewed.

Data sharing All data and material related to this research are archived and maintained according to organisational and ethical regulations. Data are not publicly available due to the risk of participant identification from specific contexts revealed when reading entire transcripts and due to the terms and conditions regarding the release of data to third parties upon which ethical approvals for this study were contingent. Reasonable requests for further information relating to data can be made to the corresponding author.

REFERENCES

1. Ramdharry G, Pollard A, Marsden J, et al. Comparing gait performance of people with Charcot-Marie-Tooth disease who do and do not wear ankle foot orthoses. *Physiother Res Int* 2012; 17:191–9.

2. Swinnen E, Lafosse C, van Nieuwenhoven J, et al. Neurological patients and their lower limb orthotics: An observational study about acceptance and satisfaction. *Prosthet Orthot Int* 2017; 4(1):41-50.

3. Vinci P and Gargiulo P. Poor compliance with ankle-foot-orthoses in Charcot-Marie-Tooth disease. *Eur J Phys Rehabil Med* 2008; 44:27–31.

4. Chisholm AE and Perry SD. Ankle-foot orthotic management in neuromuscular disorders: recommendations for future research. *Disability* 2012; 7:437–49.

5. Michael JW. KA FOs for ambulation: an orthotist's perspective. *J Prosthet Orthot* 2006; 18:187–91.

6.Chavda A and Cheema K. Analysing orthotics: availability of data and information in orthotics services in England. NHS Quality Observatory, Horley, 2014.

7. NHS England. Improving the Quality of Orthotic Services in England. 2015. https://www.england.nhs.uk/commissioning/wp-content/uploads/sites/12/2015/11/orthcs-final-rep.pdf (accessed 6th August 2019)

For peer review only - http://bmjopen.bmj.com/site/about/guidelines.xhtml

8. O'Connor J, McCaughan D, McDaid C et al. Orthotic management of instability of the knee related to neuromuscular and central nervous system disorders: systematic review, qualitative study, survey and costing analysis. *Health Technol Assess* 2016; 20 (55).

9. Swinnen E and Kerkhofs E. Compliance of patients wearing an orthotic device or orthopaedic shoes: A systematic review. *Journal of Bodywork and Movement Therapies* 2015; 19:759-70.

10. Swinnen E, Deliens T, Dewulf E et al. What is the opinion of patients with multiple sclerosis and their health care professionals about lower limb orthoses? A qualitative study using focus group discussions. *Neurorehabilitation* 2018; 42: 81-92.

11. Van der Wilk D, Hijmans JM, Postema K, et al. A user-centred qualitative study on experiences of ankle-foot orthoses and suggestions for improved design. *Prosthet Orthot Int* 2018; 42:121-128.

12. Hebert JS. Ambulatory KAFOs: a physiatry perspective. *J Prosthet Orthot* 2006; 18:169–74.

13. Peaco A, Halsne E and Hafner BJ. Assessing satisfaction with orthotic devices and services: a systematic literature review. *Am Acad Orthot Prosthet* 2011; 23(2): 95-105.

14. Pope C and Mays N. (eds.). *Qualitative research in health care*. 3rd ed. London: BMJ Books, 2006.

15. Tong A, Sainsbury P, Craig J. Consolidated criteria for reporting qualitative research (COREQ): a 32-item checklist for interviews and focus groups. *Int J Qual Health Care* 2007;19:349-57.

16. Patton MQ. *Qualitative Research and Evaluation Methods: Integrating Theory and Practice.* 4th ed. Thousand Oaks, California: Sage Publications, 2015.

17. Hennink M, Hutter I, Bailey AM. Qualitative Research Methods. London: Sage; 2011, p112.

18.Gale NK, Heath G, Cameron E, et al. Using the framework method for the analysis of qualitative data in multidisciplinary health research. *BMC Med Res Methodol* 2013; **13**:117

19. Green J and Thorogood N. *Qualitative Methods for Health Research*. 4th ed. London: Sage, 2018.

20. Richards L. *Handling Qualitative Data: A Practical Guide.* London: Sage, 2005.

21. Thorne S. Data analysis in qualitative research. *Evidence-Based Nursing* 3, 68-70.

For peer review only - http://bmjopen.bmj.com/site/about/guidelines.xhtml

22. Pope C, Ziebland S, Mays N. Analysing qualitative data. *BMJ* 2000;320 (7227):114-116.

23. McDaid C, Fayter D, Booth A, et al. Systematic review of the evidence on orthotic devices for the management of knee instability related to neuromuscular and central nervous system disorders. *BMJ Open* 2017;7:e015927. doi:10.1136/ bmjopen-2017-015927

24. Schaffalitzky E, Gallagher P, Maclachlan M, et al. Understanding the benefits of prosthetic prescription: exploring the experiences of practitioners and lower limb prosthetic users. *Disabil Rehabil* 2011; 33:1314–23.

25. Phillips MF, Radford K, Willis A. Ankle foot orthoses for people with Charcot Marie Tooth disease: views of users and orthotists on important aspects of use. *Disabil Rehabil Assist Technol* 2011; 6:491–9.

26. Fisher LR and McLellan DL. Questionnaire assessment of patient satisfaction with lower limb orthoses from a district hospital. *J Prosthet Orthot* 1989;13:29–35.

27. Bulley C, Shiels J, Wilkie K, et al. User experiences, preferences and choices relating to functional electric stimulation and ankle foot orthoses for foot drop after stroke. *Physiotherapy* 2011; 97(3): 226–33.

28. Williams A, Nester C, Ravey M. Rheumatoid arthritis patients' experiences of wearing therapeutic footwear: a qualitative investigation. *BMC Musculoskelet Disord* 2007; 8:104.

29. Van Netten JJ, Francis A, Morphet A, et al. Communication techniques for improved acceptance and adherence with therapeutic footwear. *Prosthet Orthot Int* 2017; 41(2):201-204.

30. Hutton J, Hurry M. Orthotic Service in the NHS: Improving Service Provision. Report, University of York, York Health Economics Consortium, 2009.

31. Audit Commission. Fully Equipped: The Provision of Equipment to Older or Disabled People by the NHS and Social Services in England and Wales. London: Audit Commission, 2000.

32. Hill ME and Phillips MF. Service provision for adults with long-term disability: a review of services for adults with chronic neuromuscular conditions in the United Kingdom. *Neuromuscul Disord* 2006;16:107–12.

33. Garralda ME, Muntoni F, Cunniff A, et al. Knee-ankle-foot orthosis in children with Duchenne muscular dystrophy: user views and adjustment. *Eur J Paediatr Neurol* 2006;10:186–91.

34. Saunders B, Sim J, Kingstone T, et al. Saturation in qualitative research: exploring its conceptualization and operationalization. *Qual Quant* 2018; 52:1893–1907

For peer review only

35. Hennink MM, Kaiser BN and Marconi VC. Code saturation versus meaning saturation: how many interviews are enough? *Qual Health Res* 2017; 27(4): 591-608.

36. Health Education England The future of the Orthotic and Prosthetic Workforce in England – Health Education England's Response to the NHS England Report Improving the Quality of Orthotic Services in England. 2017. https://www.hee.nhs.uk/sites/default/files/documents/Orthotic%20Report%20%20Final%20Version_0.pdf (2017, accessed 5 August 2019)

37. Brehm M, Bus SA, Harlaar J, et al. A candidate core set of outcome measures based on the international classification of functioning, disability and health for clinical studies on lower limb orthoses. *Prosthet Orthot Int* 2011; 35(3) 269–277.

38. World Health Organization (WHO) International Classification of Functioning Disability and Health: ICF 2001; Geneva: World Health Organization.

For peer review only

Figure Legends/captions

Appendix 1 Topic Guide
Appendix 2 Participants' characteristics
Figure 1 Data analysis using Framework approach
Figure 2 Study participants' perceptions of positive and negative aspects of their orthoses
Figure 3 Study participants' perceptions of positive and negative aspects of footwear

For peer review only - http://bmjopen.bmj.com/site/about/guidelines.xhtml

Figure 1 Data analysis using ‘Framework’ approach

Familiarisation with data
Reading and re-reading transcripts

Identification of codes
Identification of codes (units of meaning)
Coding scheme developed and applied to all transcripts
Data attributed to individual codes

Identification of themes
Codes combined/expanded to develop themes (broader categories of meaning)

Reviewing of themes
Iterative, cross comparative approach ensured themes supported by data
Discussion to reach consensus concerning theme development

Interpretation of findings
Exploration of relationships between themes/ interpretation of significance
Refinement of analysis through search for ‘deviant’ cases that appeared to contradict emergent findings

Illustration of theme development: Perceived positive and negative aspects of orthotic devices (Theme 3)

Examples of codes identified in the transcripts:
-Positive aspects (relating to independent mobility):
‘transformative’; *‘it changed my life’; ‘they give me independence’*

-Negative aspects (relating to poor fit/ inadequate support):
‘it doesn’t stay on my knee as I am walking’; *‘there’s not benefit in it’*; *‘it rubs your skin, I can’t wear it’*

Codes merged under unifying ‘umbrella’ theme: Perceived positive and negative aspects of orthotic devices

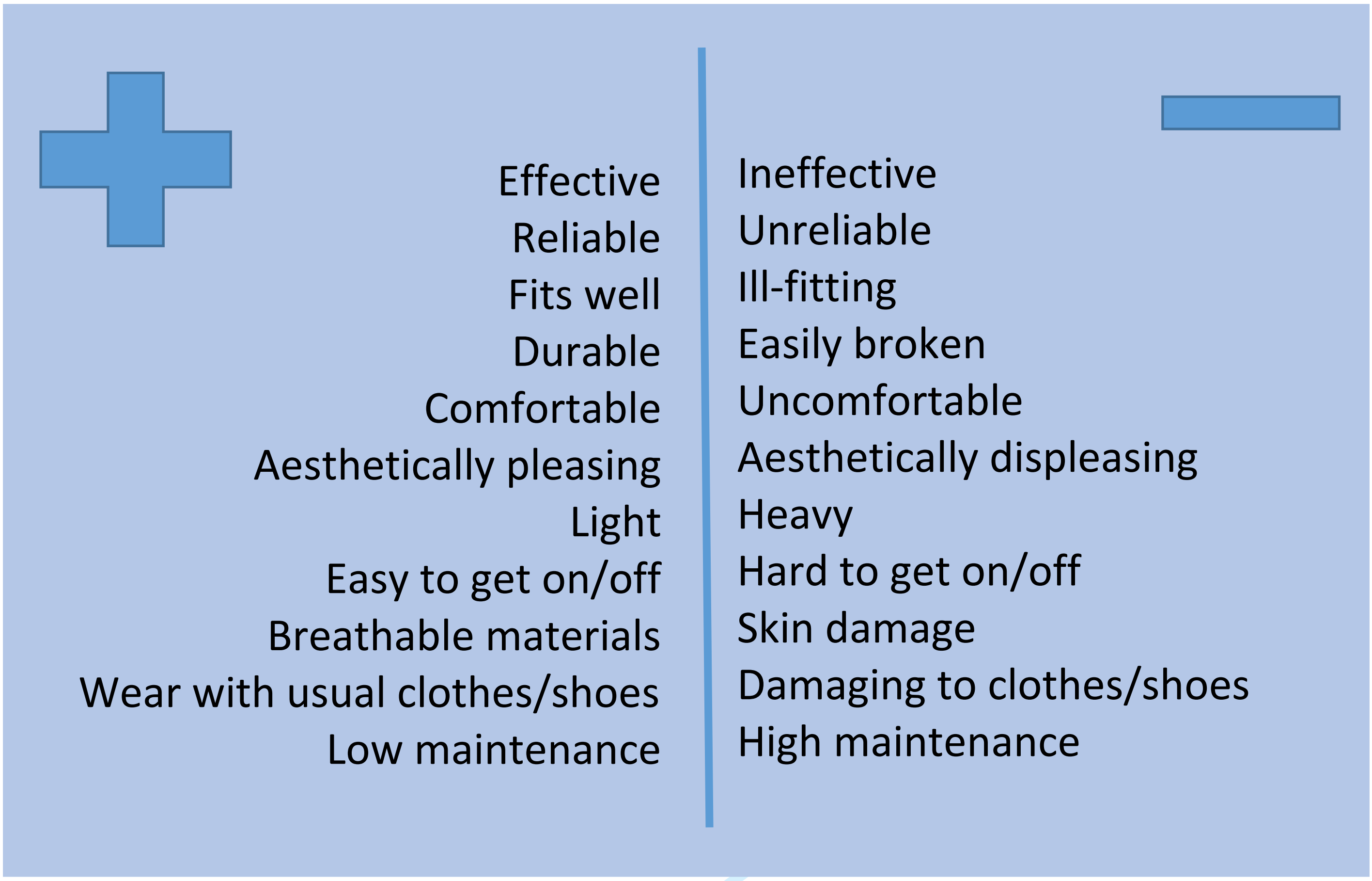

Figure 2 Study participants' perceptions of positive and negative aspects of their orthoses

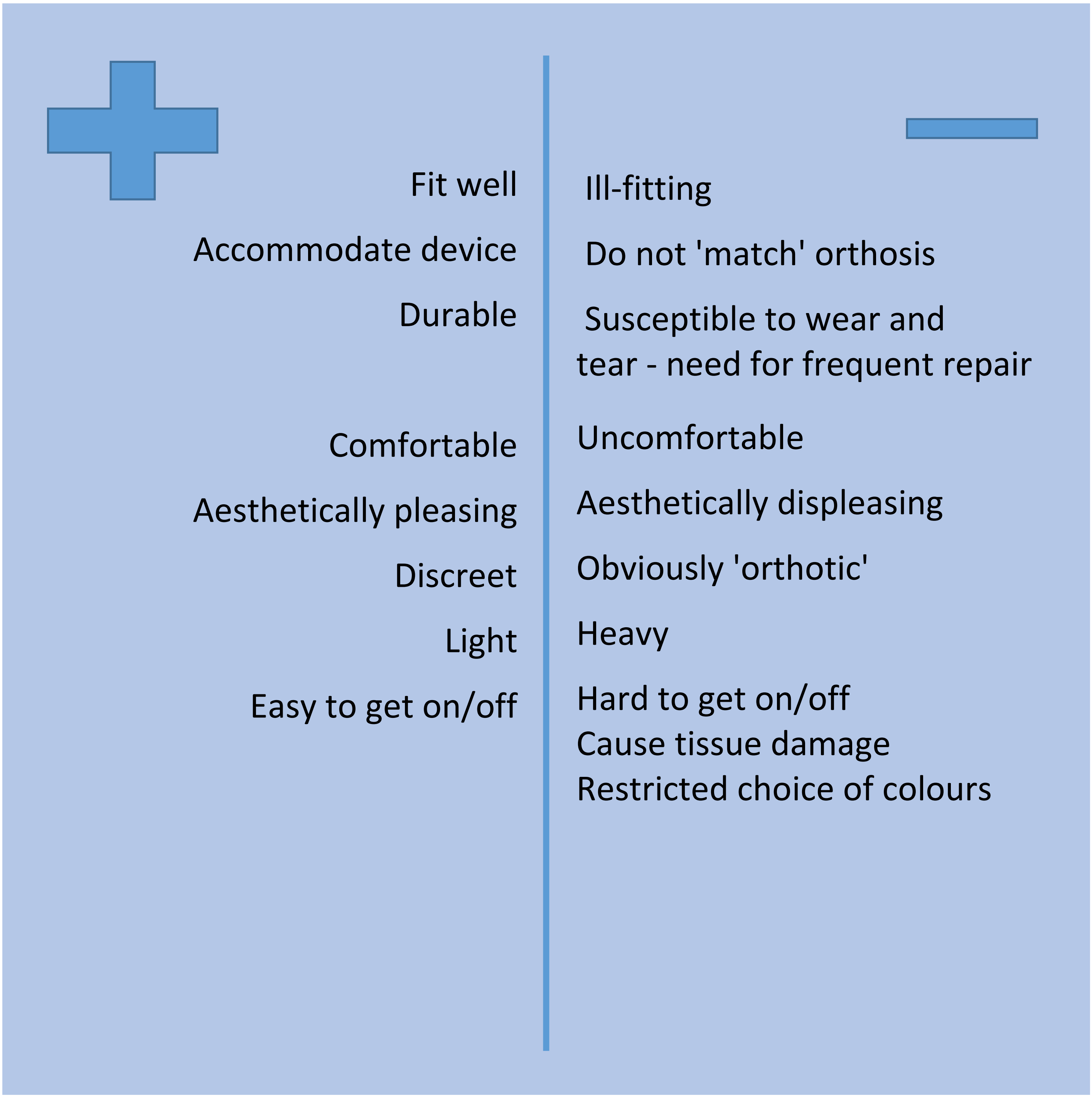

Figure 3 Study participants' perceptions of positive and negative aspects of footwear

Appendix 1 Interview Topic Guide

Researcher to ask brief details about age, occupation, ethnicity, family members, etc. to frame and contextualise the interview

-Can you tell me a little bit about yourself and how you came to have your orthotic device(s) (such as brace or callipers)? [explore important aspects further, such as diagnosis, referral, care pathway, length of time having an orthosis]

-How has the orthotic device impacted on your daily life?

-[from answer to above question] – What factors have affected you most and why? [explore important aspects further]

-What do you like, or not like, about your orthotic device? Which factors influence your decisions to use your orthotic device and why are they important?

-How effective do you think your device is in the management of knee instability?

-How do you feel about the treatment you have received in connection with your knee instability?

-How could this have been improved?

-What are the goals of treatment that matter most to you?

-How do you feel about the interactions you have had with HCPs? How could these have been improved?

-Any other aspects that have not already been discussed or you would like to expand upon?

For peer review only

Appendix 2 Participants' characteristics (study ID, age, gender, condition, duration/severity and reported device use)

Participant	Age (years)	Gender	Condition	Duration/severity/reported device use	Device
P1 (NHS)	36	Female	Poliomyelitis	Since childhood<br>Pain in knee/frequent falls<br>Devices not used as deemed ineffective and ill-fitting	KAFO and AFO
P2 (NHS)	52	Female	Spinal injury	Since childhood<br>Knee pain and instability led to difficulty balancing and falls<br>Awaiting gait assessment and referral to neuro-physiotherapy	Insert in shoe
P3 (NHS)	50	Male	Poliomyelitis	From age 5 years<br>KAFO essential for everyday mobility<br>Uses crutches to walk if not wearing KAFO	KAFO
P4 (NHS)	70	Female	Stroke	Two years previously<br>Hyperextension/pain in knee with limited mobility<br>Brace used to help with mobility but considered ill-fitting	Knee brace
P5 (NHS)	53	Male	Poliomyelitis	Since childhood<br>Severe knee pain, unable to stand for long period due to knee instability, and limited mobility prior to receiving device<br>Device described as 'transformative' in promoting mobility and independence	KAFO
P6 (NHS)	59	Female	Poliomyelitis	From age 16 months<br>Knee instability was primary problem restricting mobility<br>Device essential for mobility	KAFO
P7 (NHS)	55	Female	Multiple sclerosis	Progressive deterioration over ten years<br>Reported trips and falls and foot drop<br>Device mainly used when outdoors to assist with walking	AFO
P8 (NHS)	63	Male	Poliomyelitis	From age 2 years<br>Experienced knee instability/falls during past 5 years<br>Reported KAFO as generally offering good level of support to mobilise	KAFO and AFO

For peer review only

P9 (NHS)	67	Male	Poliomyelitis	From age 5 years<br>Experienced knee instability and falls<br>KAFO viewed as effective and essential for mobilising	KAFO
P10 (NHS)	62	Female	Multiple sclerosis	Diagnosed 30 years previously<br>Instability and weakness in knee and ankle joints, as well as foot drop, resulting in trips and falls<br>Device used mainly to assist with balance and for walking outdoors	AFO
P11 (NHS)	73	Male	Multiple sclerosis	Progressive deterioration in condition over 35 years<br>Weakness and instability in knee, foot drop, and problems with balance<br>AFO deemed effective in promoting mobility	AFO
P12 (NHS)	53	Male	Multiple sclerosis	Diagnosed 32 years previously<br>Marked deterioration in condition over past 8 years<br>Reported having experienced frequent falls, weakness in one knee and foot drop<br>AFO perceived as effective in aiding mobility	AFO
P13 (NHS)	54	Female	Multiple sclerosis	Diagnosed 5 years previously<br>Reported hyperextension of knee and foot drop, resulting in lack of balance and falls<br>Uses AFO and knee brace daily though current knee brace described as ill-fitting	AFO and knee brace
P14 (non-NHS)	64	Female	Poliomyelitis	From age 5 years<br>Reported knee hyperextension, pain and weakness, resulting in frequent trips and falls<br>Recently fitted for KAFO for first time but described it as 'cumbersome' and reported non-use as able to mobilise without it	KAFO
P15 (non-NHS)	72	Female	Poliomyelitis	From age 3 years<br>Experienced weakness in knee joint and problems with balance<br>Alternated use of one NHS-supplied and one privately purchased device	KAFO
P16 (non-NHS)	64	Male	Poliomyelitis	From age 7 years<br>Cannot stand or walk without KAFOs	KAFO (each leg) and spinal brace

For peer review only

P17 (NHS)	72	Male	Spinal injury/drop foot	Four years' duration<br>Difficulty walking on uneven ground<br>AFO perceived to promote mobility and ability to socialise	AFO
P18 (non-NHS)	73	Female	Poliomyelitis	From age 7 years<br>Developed hyperextension of knee 15 years ago and mobility deteriorated<br>KAFO not worn indoors but used for walking outdoors	KAFO
P19 (NHS)	48	Female	Spina bifida; amputation	Spina bifida since birth; date of amputation not reported<br>Severe knee weakness and instability<br>KAFO enables mobility	KAFO
P20 (NHS)	80	Male	Spinal injury	Two years' duration<br>Reported knee pain and instability and loss of balance<br>Brace deemed uncomfortable<br>Intermittent use of brace to assist with walking and employment	Knee brace
P21 (NHS)	58	Female	CMT disease	Progressive condition since birth, with marked deterioration at 30 years of age<br>Foot drop affects both feet<br>AFOs enable mobility outdoors and promote independence<br>Not used in the home	AFO
P22 (non-NHS)	63	Male	CMT disease	Progressive since birth, with marked deterioration at 40 years of age<br>Experienced knee pain and instability and 'weak' ankles, resulting in falls<br>AFOs enable mobility outdoors	AFO
P23 (NHS)	57	Male	Poliomyelitis	From age 3 years<br>Reported loss of balance, knee instability and falls<br>KAFO necessary for mobility	KAFO
P24 (NHS)	63	Male	Poliomyelitis	From age 2 years<br>Knee instability resulting in loss of balance and falls<br>Devices reported as essential for mobility - alternates use of 'new' (privately purchased) and 'old' (NHS supplied) KAFO	KAFO and AFO

Abbreviations: KAFO - knee-ankle-foot orthosis; AFO - ankle-foot-orthosis; CMT-Charcot-Marie-Tooth

For peer review only

COREQ (COnsolidated criteria for REporting Qualitative research) Checklist

A checklist of items that should be included in reports of qualitative research. You must report the page number in your manuscript where you consider each of the items listed in this checklist. If you have not included this information, either revise your manuscript accordingly before submitting or note N/A.

Topic	Item No.	Guide Questions/ Description Reported on	Page No.	Explanation
**Domain 1: Research team and reflexivity**				
**Personal characteristics**				
Interviewer/facilitator	1	Which author/s conducted the interview or focus group?	4	Dorothy McCaughan (DM) and Cath Jackson (CJ)
Credentials	2	What were the researcher's credentials? E.g. PhD, MD	n/a	(DM) MSc; BA<br>(CJ) PhD
Occupation	3	What was their occupation at the time of the study?	n/a	(DM) Research Fellow<br>(CJ) Senior Research Fellow
Gender	4	Was the researcher male or female?	n/a	Female
Experience and training	5	What experience or training did the researcher have?	4	(DM) Registered General Nurse; MSc (Nursing); BA (English)<br>(CJ) PhD; MSc; BA<br>Both researchers have previously undertaken qualitative research and have published extensively.
**Relationship with participants**				
Relationship established	6	Was a relationship established prior to study commencement?	n/a	No, participants had no relationship with the interviewer(s) prior to the study.
Participant knowledge of the interviewer	7	What did the participants know about the researcher? e.g. personal goals, reasons for doing the research?	n/a	Participants had no knowledge of the interviewer(s) prior to the study. Prior to participation in the study, participants received a patient information sheet which included details of the study and brief background information concerning the interviewer(s).
Interviewer characteristics	8	What characteristics were reported about the interviewer/ facilitator? e.g. Bias, assumptions, reasons and interests in the research topic	4	Relevant details reported in the manuscript. Data were collected by DM and CJ, who jointly analysed the data. DM and CJ each have many years' experience of applied health services research.
**Domain 2: Study design**				
**Theoretical framework**				
Methodological orientation and theory	9	What methodological orientation was stated to underpin the study? e.g. grounded theory, discourse	4	We conducted a thematic analysis of the data using 'Framework' method,

For peer review only

		analysis, ethnography, phenomenology, content analysis		underpinned by an inductive, cross-comparative approach.
**Participant selection**				
Sampling	10	How were participants selected? e.g. purposive, convenience, consecutive, snowball	4	Purposive sampling was used to identify participants across a range of conditions, age, gender, length of time fitted with an orthosis, and regions across England.
Method of approach	11	How were participants approached? e.g. face-to-face, telephone, mail, email	4	Clinical staff approached 19 patients attending NHS clinics; five people were recruited through contacts with charities/patient support groups.
Sample size	12	How many participants were in the study?	4	24 people took part in an interview for the study.
Non-participation	13	How many people refused to participate or dropped out? Reasons?	n/a	Two people were disinclined to take part in the study (due to comorbidities); no participant dropped out of the study.
**Setting**				
Setting of data collection	14	Where was the data collected? e.g. home, clinic, workplace	4	Data were collected in the homes of 21 participants; three people preferred to be interviewed by telephone.
Presence of nonparticipants	15	Was anyone else present besides the participants and researchers?	4	In two instances, the spouses/partners of participants were present and contributed to the interview.
Description of sample	16	What are the important characteristics of the sample? e.g. demographic data, date	4	Participants were adults diagnosed with a neuromuscular or CNS condition who had been offered an orthotic device for knee instability. Participant diagnoses included poliomyelitis, multiple sclerosis, Charcot-Marie-Tooth disease, spinal injury, stroke and spina bifida.
**Data collection**				
Interview guide	17	Were questions, prompts, guides provided by the authors? Was it pilot tested?	Appendix 1	A topic guide was used. This evolved as the study progressed. This is shown in Appendix 1.
Repeat interviews	18	Were repeat interviews carried out? If yes, how many?	n/a	No, repeat interviews were not conducted.
Audio/visual recording	19	Did the research use audio or visual recording to collect the data?	4	Audio recording was used to collect data.
Field notes	20	Were field notes made during and/or after the interview or focus group?	n/a	Field notes were made after the interviews, when required.

For peer review only

Duration	21	What was the duration of the interviews or focus group?	4	It varied between participants, from 45-90 minutes, with an average of about 1 hour.
aData saturation	22	Was data saturation discussed?	4	Yes, we state that data collection continued until no new information was forthcoming.
Transcripts returned	23	Were transcripts returned to participants for comment and/or correction?	n/a	No, this was not requested by any of the participants and audiotaping was clear.
**Domain 3: analysis and findings**				
**Data analysis**				
Number of data coders	24	How many data coders coded the data?	4	DM and CJ coded the data.
Description of the coding tree	25	Did authors provide a description of the coding tree?	Figure 1	The coding tree reflected the topic guide. Figure 1 provides a visual illustration of the coding and analysis of data.
Derivation of themes	26	Were themes identified in advance or derived from the data?	4/5	Themes were derived from the data, not in advance, although the topic guide provided a framework for data collection.
Software	27	What software, if applicable, was used to manage the data?	4	Data were managed using NVivo software.
Participant checking	28	Did participants provide feedback on the findings?	n/a	All participants were provided with a summary of the study findings.
**Reporting**				
Quotations presented	29	-Were participant quotations presented to illustrate the themes/findings?<br>-Was each quotation identified? e.g. participant number	5-12	Yes quotations are presented, along with the anonymised participant ID number.
Data and findings consistent	30	Was there consistency between the data presented and the findings?	5-12	Yes, we aimed to provide clarity between the data presented and the findings. Statements in text are supported by relevant quotations.
Clarity of major themes	31	Were major themes clearly presented in the findings?	5	Major themes were clearly presented with separate headings. Theme headings were summarised in Figure 2.
Clarity of minor themes	32	Is there a description of diverse cases or discussion of minor themes?	5-12	Whilst highlighting the main themes, we also incorporated a range of responses and drew attention to atypical/negative cases.

Developed from: Tong A, Sainsbury P, Craig J. Consolidated criteria for reporting qualitative research (COREQ): a 32-item checklist for interviews and focus groups. International Journal for Quality in Health Care. 2007. Volume 19, Number 6: pp. 349 – 357. Once you have completed this checklist,

For peer review only

please save a copy and upload it as part of your submission. DO NOT include this checklist as part of the main manuscript document. It must be uploaded as a separate file.

For peer review only
